# UV–Vis Spectra of Carbonic Acid: Rationalizing Experimental Redshifts between Monomer and Bulk based on (H_2_CO_3_)_n_ Calculations

**DOI:** 10.1002/cphc.202500282

**Published:** 2025-08-04

**Authors:** Dennis F. Dinu, Bastian Klein, Chaojiang Zhang, Radu A. Talmazan, Thomas Loerting, Hinrich Grothe, Ralf I. Kaiser, Maren Podewitz

**Affiliations:** ^1^ Institute of Materials Chemistry TU Wien Getreidemarkt 9/165 Vienna 1060 Austria; ^2^ W. M. Keck Research Laboratory in Astrochemistry and Department of Chemistry University of Hawaii at Manoa Honolulu Hawaii 96822 USA; ^3^ Department of Physical Chemistry University of Innsbruck Innrain 52 Innsbruck 6020 Austria

**Keywords:** carbonic acid, conformational sampling, redshift, time‐dependent density functional theory, UV–Vis

## Abstract

The UV–Vis spectra of H_2_CO_3_ are investigated in a combined experimental and theoretical approach. A sample of solid H_2_CO_3_, prepared by electron irradiation of water–carbon dioxide ice, shows characteristics of both amorphous and crystalline H_2_CO_3_ in the infrared spectrum. To rationalize the experimentally observed redshift in the UV–Vis spectra between monomer and bulk H_2_CO_3_, a systematic computational study is devised using time‐dependent density functional theory. H_2_CO_3_ is investigated from the monomer to (H_2_CO_3_)_n_ clusters, with n up to 66; in addition regular oligomer arrangements derived from previously proposed ambient‐pressure H_2_CO_3_ crystal structures are also examined. The calculations explain the UV–Vis absorption of solid carbonic acid, which is redshifted by ≈2 eV and ≈5 eV compared to the experimentally observed adiabatic ionization energy of the H_2_CO_3_ monomer. It is highlighted how these shifts emerge due to 1) increasing cluster size, 2) nonplanar arrangements, and 3) noncovalent interactions between H_2_CO_3_ chains and sheets. The study aims to establish spectrum‐to‐structure relationships and serves as computational reference data for astrochemical applications in the absence of experimental laboratory data of H_2_CO_3_ oligomers.

## Graphical abstract

In bulk carbonic acid (e.g., in interstellar icy dust grains) cluster formation produces two distinct UV‐Vis spectral features redshifted by ≈2 eV (25 nm) and by ≈5 eV (80 nm) compared to gas phase single molecule carbonic acid 's adiabatic ionization energy. Theory and experiment were used to investigate the origin of these shifts.

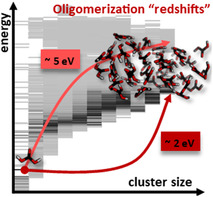

## 1 Introduction

The central molecular zone in the Milky Way with the galactic molecular cloud G+0.693‐0.027^[^ ^1^ ^]^ that harbors Sagittarius B2 is a prolific repository of complex organic species,^[^ ^2^ ^]^ where various molecules, such as aldehydes and alcohols, were found via radio spectroscopy.^[^ ^3^ ^]^ In 2023, the *cis–trans* conformer of carbonic acid (ct‐H_2_CO_3_) was observed in G+0.693‐0.027.^[^ ^4^ ^]^ This is a higher energy, more polar conformer than the more stable *cis–cis* conformer of the carbonic acid. The detection is particularly impressive because it proves the stability of H_2_CO_3_ in the absence of water.^[^ ^5^ ^]^ On the other hand, the detection also demonstrates the need for accurate laboratory reference data to uniquely identify complex organic molecules in the interstellar medium by spectroscopic techniques.^[^ ^4^ ^]^

**Figure 1:**
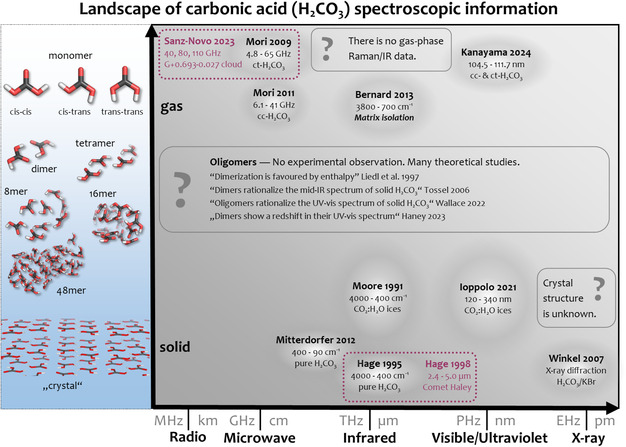
Available laboratory spectroscopic data for carbonic acid (H_2_CO_3_). The *y*‐axis illustrates structural motifs of H_2_CO_3_ from the ordered structure (**crystal**) in the solid, over multiple interacting molecules (**oligomers**) to the single molecule (**monomer**) in the gas‐phase or matrix isolation. The *x*‐axis shows regions of the electromagnetic spectrum. The white spots in the plot highlight laboratory experimental observations of H_2_CO_3_ in the respective phase and spectral region. Astrochemical detections are highlighted in purple. Regions that are not yet experimentally accessed are highlighted with a question mark. There are no gas‐phase IR or Raman data of oligomers, and the crystal structure at ambient pressure is unknown.

The detection of H_2_CO_3_ comes relatively late compared to other, more complex molecules. This is due to the limited availability of laboratory reference data in several spectral ranges for H_2_CO_3_. **Figure** **1** highlights the status quo of H_2_CO_3_ spectroscopic data in various regions of the electromagnetic spectrum. Our overview includes available laboratory and computational data for H_2_CO_3_ as a single molecule in the gas phase, for H_2_CO_3_ clusters, as well as for crystalline and amorphous solids. At first glance, it becomes evident that most spectral information is available in the infrared (IR) and the ultraviolett–visible (UV–Vis) region, with a focus on the solid H_2_CO_3_. In the following, we elaborate on the available data in terms of (A) regions in the electromagnetic spectrum from radio waves to X‐rays and (B) structural motifs of H_2_CO_3_ from the gas phase to the solid.

Within this broader context, the present study addresses the phenomenon we refer to as the **redshift relative to the monomer**. This describes a shift of UV–Vis absorption bands to lower energies observed when comparing molecular aggregates or the condensed phase to isolated monomers. It does not reflect a shift of a single electronic transition. Instead, it arises from the cumulative influence of intermolecular interactions on multiple electronic states. Although this is a figurative use of the term “redshift,” it is adopted in the literature as a convenient shorthand for this spectral behavior.^[^ ^6^ ^]^

### 1.1 Spectroscopic Signatures of H_2_CO_3_

#### 1.1.1 Radio and Microwave Region

In astro‐spectroscopy, it is common to search for molecules via the radio to microwave (MW) and millimeter wave (mm) region.^[^ ^7^ ^]^ Regarding this region, Mori et al. (cf. Mori 2009 in Figure 1) performed laboratory experiments in a gas‐phase supersonic jet of H_2_CO_3_ to obtain MW/mm spectra of ct‐H_2_CO_3_ from 4.8 to 65 GHz and of *cis–cis*‐H_2_CO_3_ (cc‐H_2_CO_3_) from 6.1 to 41 GHz.^[^ ^8^, ^9^ ^]^ Based on such laboratory reference data, Sanz‐Novo et al. (cf. Sanz‐Novo 2023 in Figure 1) detected ct‐H_2_CO_3_ in the G+0.693‐0.027 galactic molecular cloud for MW spectra around 40 GHz and indirectly for mm spectra around 90 GHz and 110 GHz, using the ground‐based observatories Yebes 40 m and IRAM 30 m.^[^ ^4^ ^]^ This detection of H_2_CO_3_ via MW spectroscopy is a prime example of the fruitful interplay of laboratory experiments and astro‐observatory data.

In the sub‐millimeter (sub‐mm) and far infrared (far‐IR) regions, there are no specific studies on gas‐phase H_2_CO_3_. However, laboratory spectroscopy in this region is generally sparse (cf., terahertz gap^[^ ^7^ ^]^). For solid H_2_CO_3_, the low frequency‐shift Raman spectrum (<500 cm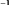) gives some insights on the far‐IR region^[^ ^10^ ^]^ (cf. Mitterdorfer 2012 in Figure 1). However, the mid‐infrared (mid‐IR) region of solid H_2_CO_3_ is more widely studied. This is because mid‐IR was often used to demonstrate the existence of H_2_CO_3_ from various synthesis routes.

#### 1.1.2 Mid‐IR and UV–Vis Region

In 1991, Moore et al. (cf. Moore 1991 in Figure 1) observed mid‐IR features of H_2_CO_3_ by proton irradiation of CO_2_:H_2_O ice mixtures.^[^ ^11^ ^]^ Multiple irradiation experiments confirmed the existence of H_2_CO_3_, by irradiating ices containing pure CO_2_ or mixtures thereof with protons,^[^ ^12^, ^13^, ^14^, ^15^, ^16^ ^]^ helium ions,^[^ ^14^, ^16^, ^17^, ^18^ ^]^ electrons,^[^ ^19^, ^20^, ^21^, ^22^ ^]^ and larger ions.^[^ ^23^, ^24^ ^]^ Some studies also demonstrated that UV photolysis of CO_2_:H_2_O ice mixtures produces H_2_CO_3_.^[^ ^15^, ^25^, ^26^ ^]^ In most ice irradiation experiments, the IR spectrum of solid H_2_CO_3_ was studied, while UV–Vis spectra have been scarcely investigated.^[^ ^20^, ^22^, ^26^ ^]^ However, as these irradiation experiments produce multiple side products, assigning all H_2_CO_3_ bands is impossible.

In 1993, Hage et al. observed the methyl ester of H_2_CO_3_ by protonating bicarbonates at cryo‐conditions in methanolic solution,^[^ ^27^ ^]^ and in 1995, they observed H_2_CO_3_ in aqueous solution^[^ ^28^ ^]^ (cf. Hage 1995 in Figure 1). This first synthesis of pure solid H_2_CO_3_ gave rise to the first thorough assignments of H_2_CO_3_ in the mid‐IR region. Based on their mid‐IR spectra, Hage et al.^[^ ^29^ ^]^ (cf. Hage 1998 in Figure 1) suggested that some spectral features of solid H_2_CO_3_ can be compared to unassigned bands in the spectrum of Halley's Comet.^[^ ^30^ ^]^ Their synthesis procedure was thoroughly investigated^[^ ^10^, ^18^, ^31^, ^32^, ^33^, ^34^, ^35^ ^]^ and eventually led to sublimation experiments that have allowed matrix‐isolation IR experiments of the H_2_CO_3_ monomers.^[^ ^36^, ^37^, ^38^, ^39^, ^40^, ^41^ ^]^

The different H_2_CO_3_ synthesis routes, the presence of contaminants, and the differences between amorphous and crystalline phases, or simply temperature effects, led to various shifts in the vibrational frequencies: Taking the characteristic *ν*C=O stretching as an example, the IR spectra from irradiation synthesis routes yield wavenumbers between 1700 and 1730 cm^−1^.^[^ ^11^, ^14^, ^15^, ^19^, ^25^ ^]^ In the IR spectra from the acid–base synthesis,^[^ ^28^ ^]^ the band is at 1700 cm^−1^. Matrix isolation of the H_2_CO_3_ monomer shows the *ν*C=O stretching at 1790 cm^−1^ (cf. Bernard 2013 in Figure 1),^[^ ^37^, ^39^ ^]^ which compares well with anharmonic molecular vibration computations.^[^ ^42^ ^]^ It is worth mentioning that in their 2007 paper, Winkel et al. reported the X‐ray diffraction pattern of the H_2_CO_3_/KBr binary mixture (cf. Winkel 2007 in Figure 1),^[^ ^34^ ^]^ while X‐ray data on the “pure” H_2_CO_3_ at ambient pressure is still lacking.

While the mid‐IR region is well known for the solid and matrix‐isolated species, it was only partially investigated for the aqueous solution,^[^ ^43^, ^44^ ^]^ and the gas‐phase observation remains an open challenge. Such lack of experimental data can be compensated by calculations, which is essential for spectroscopic observations, e.g., from the James Webb Space Telescope.^[^ ^45^ ^]^ Ioppolo et al. suggested that detection of H_2_CO_3_ in the UV–Vis region is more likely than in the IR region, where interference with more abundant species complicates its identification.^[^ ^22^ ^]^ However, the laboratory data in the UV region are sparse, too.^[^ ^46^ ^]^

In 2014, Jones et al. indicated some UV features in their study on H_2_O:CO_2_ ices. The first crude assignment to H_2_CO_3_ for a feature at 200 nm (6 eV) was later given by Parivataa.^[^ ^20^ ^]^ Ioppolo et al. produced H_2_CO_3_ by electron irradiation (1 keV) of CO_2_:H_2_O ice mixtures and observed it through vacuum UV photoabsorption (cf. Ioppolo 2021 in Figure 1).^[^ ^22^ ^]^ They assigned two bands to two H_2_CO_3_ polymorphs in the 139 nm (9 eV) to 200 nm (6 eV) region. In contrast to mid‐IR and MW assignments (see above), the UV–Vis region of solid H_2_CO_3_ lacks a profound theoretical understanding of the observed transitions.

Only recently, Kanayama et al.^[^ ^47^ ^]^ (cf. Kanayama 2024 in Figure 1) successfully generated H_2_CO_3_ via flash pyrolysis^[^ ^39^ ^]^ and characterized it using mass‐selected threshold photoelectron spectroscopy with vacuum UV synchrotron radiation. Their study provided precise adiabatic ionization energies (AIEs) of the cc‐H_2_CO_3_ and ct‐H_2_CO_3_ conformers, corresponding to transitions near 110.0 nm (11.27 ± 0.02 eV) and 110.9 nm (11.18 ± 0.03 eV), respectively. While these values represent the most accurate data available for the energetic threshold of electronic excitation in the single molecule, they pertain to ionization processes rather than neutral excitations. The extent to which Rydberg or valence excited states contribute to the absorption spectrum near this energetic region remains unresolved.

### 1.2 Structural Motifs of H_2_CO_3_

Besides the irradiation of ice mixtures, first shown by Moore et al.,^[^ ^11^ ^]^ the acid–base synthesis introduced by Hage et al.,^[^ ^27^ ^]^ and the gas‐phase pyrolysis by Reisenauer et al.,^[^ ^39^ ^]^ other synthesis routes have been suggested. Oba et al.^[^ ^48^ ^]^ proposed the reaction of CO with 2 OH radicals: CO + 2OH → H_2_CO_3_. Wang et al. proposed to prepare solid H_2_CO_3_ by heating CO_2_:H_2_O mixtures at high pressure with a CO_2_ laser.^[^ ^49^ ^]^ Recently, Berni et al. demonstrated a synthetic route to obtain two distinct carbonic acid crystals from the fast, cold compression of pristine clathrate hydrate samples.^[^ ^50^ ^]^ This amount of diverse synthesis routes can be expected to produce further spectral data in the future. The spectrum‐to‐structure relationship is essential for developing a chemical model based on the spectral signatures. In the following, we summarize the current state of the art and highlight gaps in assigning spectrum‐to‐structure relationships for H_2_CO_3_ monomers, oligomers, and both crystalline and amorphous structures.

#### 1.2.1 Monomer

Monomers predominate gas‐phase experiments, and calculations suggested three possible conformers: cc‐H_2_CO_3_, ct‐H_2_CO_3_, and *trans*,*trans*‐H_2_CO_3_ or tt‐H_2_CO_3_ (cf. “monomer” in Figure 1). The cc‐H_2_CO_3_ conformer is the lowest in energy, followed by the ct‐H_2_CO_3_ and tt‐H_2_CO_3_ conformers.^[^ ^51^ ^]^ In MW experiments, the ct‐H_2_CO_3_ was easier to observe due to its higher dipole moment.^[^ ^4^, ^8^, ^9^ ^]^ In UV–Vis experiments, the cc‐H_2_CO_3_ and ct‐H_2_CO_3_ conformers were simultaneously observed.^[^ ^47^ ^]^ To our knowledge, no gas‐phase IR spectra of H_2_CO_3_ monomers are available. However, the monomer was the main species observed through matrix‐isolation IR spectroscopy,^[^ ^37^, ^39^ ^]^ both cc‐H_2_CO_3_ and ct‐H_2_CO_3_ conformers. Note that UV–Vis spectra of matrix‐isolated H_2_CO_3_ in rare gases have not been studied yet.

#### 1.2.2 Oligomers

To the best of our knowledge, no experimental data are currently available for H_2_CO_3_ oligomers. While dimers were identified in matrix isolation studies of the methyl hemiester of carbonic acid,^[^ ^41^ ^]^ no such dimers were found by the same authors in matrix isolation studies of carbonic acid itself. In addition, no oligomers were identified in gas‐phase experiments regardless of the electromagnetic region studied. This lack can probably be attributed to the difficulty of stabilizing species consisting of a selected number of H_2_CO_3_.

In contrast, various computational studies investigated H_2_CO_3_ clusters (cf. “dimer”, “tetramer”, etc. in Figure 1). Ab initio energy calculations indicate that enthalpy favors dimerization.^[^ ^52^, ^53^ ^]^ For oligomer structures with up to 5 H_2_CO_3_ units, planar chains of hydrogen‐bonded structures were considered to be more stable than 3D structures.^[^ ^54^ ^]^ In the IR spectra of solid H_2_CO_3_, typical spectral features of the monomer are not observed. Instead, shifts in the *ν*O—H and *ν*C=O stretching vibrations to lower wavenumbers due to hydrogen bonding in the dimer help to better understand the experimental observation.^[^ ^53^, ^55^, ^56^ ^]^ A systematic evaluation of around 40 dimer variations suggests that hydrogen‐bonded, planar structures are the most stable.^[^ ^57^, ^58^ ^]^

#### 1.2.3 Solids

Solid H_2_CO_3_ can be amorphous or crystalline, while mixtures of both are also possible. While crystalline phases can be resolved once a crystal structure becomes available (see below), amorphous solids are more difficult to model because there are numerous possibilities for building amorphous H_2_CO_3_ with different relative energies. Consequently, until today, no atomistic extended structural model for amorphous H_2_CO_3_ exists that can be used to establish a structure‐to‐spectrum relationship.

Mid‐IR spectra, recorded for solid H_2_CO_3_, can be reasonably assigned by comparison with calculations of isolated molecules.^[^ ^55^ ^]^ In contrast, experimental UV–Vis features are much more challenging to interpret, and it remains unclear which structural motifs to expect for solid H_2_CO_3_.

Recently, Wallace et al. attempted to establish a first spectrum‐to‐structure relationship and calculated UV–Vis features for helical and linear H_2_CO_3_ oligomers^[^ ^59^ ^]^ and later nonlinear oligomers,^[^ ^60^ ^]^ which they correlated with experimentally observed UV–Vis features observed by Ioppolo et al.^[^ ^22^ ^]^ From equation of motion coupled cluster singles doubles (EOM‐CCSD) calculations, it was suggested that redshifts in the UV–Vis spectrum, referenced to the monomer, occur from perpendicular arrangement of pairs of dimers^[^ ^60^ ^]^ in an equilibrium structure. However, later work of the same authors attributed the redshift to nonequilibrium structures that resulted from the out‐of‐plane distortion of the *τ*(OCOO) dihedral.^[^ ^6^ ^]^ Given the low dihedral potential, the authors suggested that such nonequilibrium structures might be frozen out in H_2_CO_3_ ices; however, this claim remains open to further validation.

These works demonstrate the sensitivity of UV–Vis experiments to the underlying structure and emphasize the importance of reliable structural models to obtain meaningful results. Hence, it is necessary to investigate the spectrum‐to‐structure relationship further using a systematic approach that goes from monomers to oligomers. While computational costs of the CCSD approach limit studies to small oligomers such as dimers, time‐dependent density functional theory (TD‐DFT) is suitable for calculating electronic excitation spectra, justifying investigations of higher oligomers.^[^ ^61^ ^]^

Modeling oligomer structures to interpret the spectroscopic information of solid H_2_CO_3_ is a reasonable approximation for amorphous solids of nonordered structural motifs. However, assuming the solid to be crystalline, the spectra would be ideally modeled based on ordered structural motifs. For this purpose, an experimental crystal structure would be ideal. While neutron diffraction suggested a monoclinic *P*2_1_/c crystal structure of D_2_CO_3_ at high‐pressure (1.85 GPa),^[^ ^62^ ^]^ the crystal structure of H_2_CO_3_ at ambient pressure remains unknown.

Winkel et al. reported powder X‐ray diffraction data of amorphous and crystalline H_2_CO_3_.^[^ ^34^ ^]^ However, the thin film nature of the sample and the presence of interfering Bragg peaks from the by‐phase KBr have impeded the determination of the ambient‐pressure crystal structure of H_2_CO_3_. They proposed several hydrogen‐bonding motifs for hypothetical low‐energy crystal structures by leveraging crystal structure prediction methods (cf. “crystal” in Figure 1).^[^ ^34^ ^]^ In comparison with Raman/IR spectra, centrosymmetric building blocks were suggested for the crystal.^[^ ^10^ ^]^ From calculations, Reddy et al. demonstrated that chainlike topologies ( *P*2_1_/c and *C*2/c space group) are more stable than sheet‐like hydrogen‐bonding networks, with the *C*2/c crystal being marginally more stable, in agreement with experimental powder X‐ray patterns.^[^ ^63^, ^64^ ^]^

To summarize, identifying the underlying structural features of H_2_CO_3_ that explain experimental spectroscopic findings remains challenging. The bulk of experimental data in any region of the electromagnetic spectrum relates to the solid, for which structural information is missing, both for the crystalline and the amorphous solids.

There is no easily discernible spectrum‐to‐structure relationship for large amorphous structures because they consist of many slightly different structural subunits that must be modeled. Hence, it can be instructive to systematically study the impact of H_2_CO_3_ oligomer size and topology on spectral properties to investigate spectral trends regarding the structural motifs. Wallace and Fortenberry have pioneered significant work in this direction by investigating clusters, including non energy‐minimum structures.^[^ ^6^, ^59^, ^60^, ^65^, ^66^ ^]^ However, a systematic study starting from the H_2_CO_3_ monomer and successively investigating larger oligomers is missing. Such a study could contribute to the spectrum‐to‐structure understanding of the UV–Vis spectrum of H_2_CO_3_ and provide important computational reference data for further laboratory studies and astrochemical sensing.

In this work, we present an IR and UV–Vis investigation of solid H_2_CO_3_, focusing on determining whether it is amorphous or crystalline. To aid in interpreting experimental findings, we calculate UV–Vis spectra as reference data using TD‐DFT to assess the impact of oligomer size on the spectra and compare the results with available laboratory characterizations of H_2_CO_3_ and its isotopologues. To determine the structural diversity of amorphous structures, we performed extensive conformer searches and investigated cluster size effects in regular slabs derived from proposed H_2_CO_3_ structures. This work systematically shows the emergence of spectral shifts in the spectrum due to increasing oligomer size, decreasing planarity, and noncoplanar arrangements. All conformers show similar spectra for the largest oligomer clusters, meaning that in all clusters, similar structural elements are found that give rise to the same spectral features.

## 2 Results and Discussion

### 2.1 Solid H_2_CO_3_ from Ice Mixtures

We synthesized H_2_CO_3_ from irradiated H_2_O and CO_2_ ice mixtures in an ultra‐high vacuum setup at 5 K. Mass spectrometry and UV–Vis spectroscopy monitored the process. IR spectroscopy confirmed H_2_CO_3_ formation with a column density of 1.97 × 10^16^ molecule cm^−2^ and a thickness of 20 ± 2 nm.

#### 2.1.1 IR Spectroscopy

**Figure 2:**
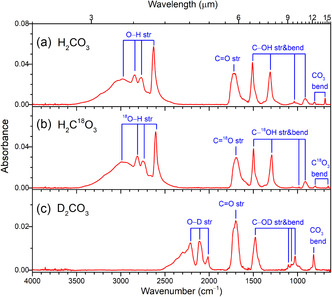
Fourier transform infrared spectra of solid carbonic acid recorded at 5 K: a) H_2_CO_3_, b) H_2_C^18^O_3_, and c) D_2_CO_3_. The H_2_CO_3_ products are from 5 keV electron irradiated water and carbon dioxide ice mixtures at 5 K, followed by heating to 210 K and recooling to 5 K. Absorption bands from carbonic acid are marked. The abbreviation str refers to stretch, while bend refers to a bending motion.

The IR spectra of the three isotopologues investigated here (H_2_CO_3_, H_2_C^18^O_3_, and D_2_CO_3_ ) are shown in **Figure** **2**, and the corresponding assignments are given in **Table** **1**. We consider these spectra to investigate (A) whether our nanometer film of H_2_CO_3_ is amorphous or crystalline and (B) the magnitude of IR redshifts from the monomer to the bulk by comparison with IR reference data for the monomer.

**Table 1:** Infrared absorptions for carbonic acid (H_2_CO_3_) produced from 5 keV electron irradiated water and carbon dioxide ice mixtures at 5 K, along with those of isotopic molecules H_2_C^18^O_3_ and H_2_CO_3_ obtained under the same conditions.

Position [cm^−1^]^a^^)^			Assignment^b^^)^
H_2_CO_3_	H_2_C^18^O_3_	D_2_CO_3_	
3500–2500	3500–2500	2500–1900	νO—H
1726	1696	1700	νC=O
1510	1498	1478	ν_as_C—OH
1310	1292	1028	δ_ip_C—OH
1038	983	1104/1072	ν_s_C—OH
912	910		δ_oop_C—OH
812	796	819	δ_oop_CO_3_
688	655		δ_ip_CO_3_

a) All the irradiated ices were heated to 210 K and cooled to 5 K to sublimate the water and carbon dioxide.

b) The assignments are in line with literature references: DelloRusso et al.;^[^ ^13^ ^]^ Gerakines et al.,^[^ ^15^ ^]^ Zheng and Kaiser,^[^ ^19^ ^]^ Hage et al.;^[^ ^31^ ^]^ Oba et al.,^[^ ^48^ ^]^ Bernard et al.;^[^ ^37^ ^]^ Bernard et al.^[^ ^36^ ^]^ The labels *ν* denote stretching vibrations and *δ* bending vibrations, with the subscripts s/as denoting a symmetric or anti‐symmetric vibration and ip/oop an in‐plane or an out‐of‐plane vibration.

(A) Generally, our IR spectra match the observations of solid H_2_CO_3_.^[^ ^27^, ^28^, ^36^ ^]^ Winkel et al.^[^ ^34^ ^]^ studied spectral changes in the IR region of H_2_CO_3_ from fully amorphous to fully crystalline. Compared to the IR spectra by Winkel et al., our IR spectra in Figure 2 show mostly features of amorphous, or a‐H_2_CO_3_, but also some indications of crystalline, or c‐H_2_CO_3_. This can be observed for all spectral regions: (νO—H region) c‐H_2_CO_3_ has a double peak at 2750/2830 cm^−1^, while a‐H_2_CO_3_ shows a single broad feature. We observe the double peak. (νC=O region) a‐H_2_CO_3_ has a feature at 1700 cm^−1^ with two shoulders at 1745 cm^−1^/1665 cm^−1^ for c‐H_2_CO_3_, which are broad with a maximum at 1720 cm^−1^ and a shoulder at 1630 cm^−1^ for a‐ and partly a‐H_2_CO_3_. We observe a similar formation in our spectrum. (ν_as_C—OH region) a‐H_2_CO_3_ has a band at 1476 cm^−1^. c‐H_2_CO_3_ has a band at 1501 cm^−1^. We observe both bands. a‐H_2_CO_3_ has a low‐intensity shoulder near 1370 cm^−1^, which disappears after crystallization. We observe this low‐intensity shoulder. (ν_s_C—OH region) c‐H_2_CO_3_ has a sharp band at 1035 cm^−1^. a‐H_2_CO_3_ has a broader feature at 1020 cm^−1^. We observe the sharp band on top of the broader feature. (δ_oop_C—OH region) c‐H_2_CO_3_ has a double peak at 900/880 cm^−1^. a‐H_2_CO_3_ has a single broad band. We observe the broad feature only. (δ_ip_CO_3_ region) c‐H_2_CO_3_ has a double peak at 683/660 cm^−1^. a‐H_2_CO_3_ has single band at 650 cm^−1^. We observe a rather sharp band at ≈680 cm^−1^, but there is no second band.

(B) To quantify the IR redshift from the monomer to the bulk, we consider the assignment of the νO—H vibration and the νC=O vibration. The νO—H vibration can be associated with the complex multipeak pattern in the region from 3500 to 2500 cm^−1^, making an assignment rather superficial. These peaks arise from various O—H stretching vibrations due to the solid's numerous hydrogen‐bonded OH groups. With the monomer νO—H stretching located most likely at around 3650 cm^−1^ (cf., latest matrix‐isolation IR assignment by Schlagin et al.^[^ ^42^ ^]^), the IR redshift from the monomer to the bulk is between 150 and 1150 cm^−1^. On the energy scale this corresponds to a shift between 19 and 144 meV.

A more precise derivation of the IR redshift from the monomer to the bulk can be made for the νC=O stretching. In this regard, we may reconsider various previous experiments from the literature. From irradiation (700 keV H^+^) of H_2_O:CO_2_ ice mixtures, Moore et al.^[^ ^11^ ^]^ initially observed the νC=O vibration at 1702 cm^−1^. Later, Brucato et al.^[^ ^14^ ^]^ observed it at 1713 cm^−1^ (3 keV He^+^) and 1726 cm^−1^ (1.5 keV H^+^) when irradiating H_2_O:CO_2_ ice mixtures, and at 1727 cm^−1^ (1.5 keV H^+^) when irradiating pure CO_2_ ice. Zheng et al.^[^ ^19^ ^]^ report the band at 1723 cm^−1^ when irradiating H_2_O:CO_2_ ice mixtures with 5 keV electrons. In their UV photolysis experiments, Gerakines et al.^[^ ^15^ ^]^ (and later Wu et al.^[^ ^25^ ^]^) observe the band at 1719 cm^−1^. All these “irradiation experiments” yield IR spectra influenced by hydrogen bonds with contaminations such as water. In the acid–base synthesis of pure H_2_CO_3_ by Hage et al.,^[^ ^28^ ^]^ the νC=O vibration can be assigned to a band at 1700 cm^−1^, which is only shifted due to hydrogen bonds between multiple H_2_CO_3_ units. From acid–base reaction synthesized H_2_CO_3_ isolated in argon matrix, Bernard et al.^[^ ^37^ ^]^ assigned the νC=O stretching of the monomer to 1790 cm^−1^. Reisenauer et al.^[^ ^39^ ^]^ confirmed this observation in the argon matrix, using gas‐phase pyrolysis to synthesize H_2_CO_3_. We observe a band at 1726 cm^−1^, which best agrees with the observations by Brucato et al.,^[^ ^14^ ^]^ suggesting hydrogen bonds with water contamination. Relying on the matrix‐isolation IR spectra, the monomer νC=O stretching is most likely at around 1790 cm^−1^. These observations are likely prone to matrix shifts.^[^ ^67^ ^]^ The gas‐phase monomer has not yet been observed in IR. Hence, the matrix shift can only be inferred by comparing the calculated IR spectra of the single molecule. The latest anharmonic calculations suggest an argon matrix shift of 10 cm^−1^.^[^ ^42^ ^]^ Thus, the redshift for νC=O in the IR spectrum from the monomer to the solid is around 70 cm^−1^. On the energy scale this corresponds to a shift of ≈8.7 meV. The shallow IR redshifts in the meV region are convenient from a theoretical perspective. A theoretical model for the monomer vibrations yields wavenumbers close to what can be observed in the solid IR spectrum, especially in the IR region below 2000 cm^−1^. In other words, the structure‐to‐spectrum relationship for the monomer can be used to interpret a solid IR spectrum. Successful assignments in the IR spectra of solids containing H_2_CO_3_ are possible without modeling the bulk.

#### 2.1.2 UV–Vis Spectroscopy

**Figure 3:**
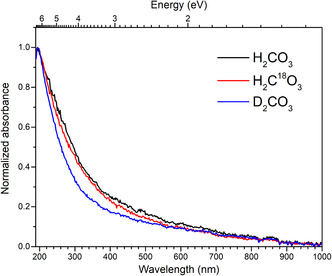
UV–Vis spectra of solid carbonic acid (H_2_CO_3_, black line). The H_2_CO_3_ is synthesized by processing water and carbon dioxide ice mixtures upon 5 keV energetic electrons at 5 K, then heated to 210 K, and recooled to 5 K. The spectra of isotopologues H_2_C^18^O_3_ (red line) and D_2_CO_3_ (blue line) obtained under the same conditions are also presented. All spectra are normalized to the 194 nm (6 eV) peak.

For the UV experiments, we used a rhodium‐coated silver mirror with enhanced UV reflectance, and ice deposition was monitored via interferometry. The ice, 850 ± 50 nm thick, was irradiated with 5 keV electrons, depositing 47.6 ± 5.0 eV amu^−1^, and then heated to 210 K to desorb reactants. The resulting UV–Vis spectra from such prepared H_2_CO_3_ are shown in **Figure** **3**. In contrast to the IR spectra, the UV–Vis spectra of the different isotopologues are very similar. We observe a slight decrease in band broadening from the “light” H_2_CO_3_ to the “heavier” isotopologues. Our UV–Vis spectra of solid H_2_CO_3_ show one spectral feature at 194 nm (6 eV) that agrees with the observation of a spectral feature at 200 nm (6 eV) by Pavithraa et al.^[^ ^26^ ^]^ Ioppolo et al.^[^ ^22^ ^]^ observed a similar band at 200 nm (6 eV) and an additional feature at 139 nm (9 eV).

Recently, Kanayama et al. reported gas‐phase data for H_2_CO_3_ monomers, determining AIEs near 113 nm (11 eV), using vacuum UV ms‐TPE spectroscopy.^[^ ^47^ ^]^ While these AIEs represent accurate energetic thresholds for ionization, they do not correspond to direct neutral electronic excitations. Nonetheless, they provide an important upper bound for absorption. Comparing this monomer reference to bulk solid H_2_CO_3_, we observe two distinct redshifts on the energy scale: a shift of ≈5 eV with reference to our UV–Vis measurements, and a shift of ≈2 eV, with reference to the additional feature reported by Ioppolo et al. in their vacuum ultra violet (VUV) absorption study.^[^ ^22^ ^]^ These redshifts, compared to the AIE of the monomer, reflect the influence of clustering, solid‐state effects, and intermolecular interactions on the electronic structure of carbonic acid.

These considerations demonstrate another contrast to IR spectroscopy. The redshift from monomer to bulk is much more prominent in the UV–Vis region than in the IR region. Comparing the magnitude of the redshifts illustrates the different demands on spectrum‐to‐structure relationships in the IR and UV–Vis regions. In the UV–Vis region, the theoretical model for the electronic excitations in the monomer yields transition energies far away from what can be observed in the solid UV–Vis spectra. For UV–Vis spectroscopy, the structure‐to‐spectrum relationship derived for the monomer cannot be directly used to interpret the solid. The theoretical model must consider various structural motifs to rationalize the above‐mentioned UV redshifts.

### 2.2 Simulated UV–Vis Spectra

We simulated the UV–Vis spectra of H_2_CO_3_ oligomers with increasing size using time‐dependent density functional theory (TD‐DFT). These approximate calculations can be expected to come with a certain discrepancy compared to the experiment. Wallace demonstrated that the difference between TD‐DFT and more accurate EOM‐CCSD calculations is state‐dependent and in the ±0.5 eV range.^[^ ^66^ ^]^ Hence, a constant shift from EOM‐CCSD as a correcting factor for our TD‐DFT calculations may not be applied.

Nonordered structural motifs were derived from conformational sampling of the potential energy surface to mimic excerpts from amorphous solids. Energetically favorable conformers were identified using the Conformer–Rotamer Ensemble Sampling Tool (CREST).^[^ ^68^ ^]^ This approach captures the structural diversity of the clusters, allowing the identification of the energetically most stable structures as cluster size increases. The structural diversity then enables the evaluation of how different arrangements influence the energy and UV–Vis spectra. Due to the known shortcomings of the semiempirical GFN2‐xTB utilized for the CREST calculations, benchmarks against conventional DFT were conducted (Figure S1–S2 in the Supporting Information).

Additionally, slabs with an increasing number of H_2_CO_3_ units were extracted from previously proposed periodic crystal structures with ordered structural motifs.^[^ ^34^ ^]^ The spectral ranges of these species were compared to analyze shifts in absorption band maximum compared to the experimentally observed adiabatic ionization energies (AIE) of the H_2_CO_3_ monomer as a function of cluster size, which is referred to as redshift.

#### 2.2.1 Monomer and Dimer

**Figure 4:**
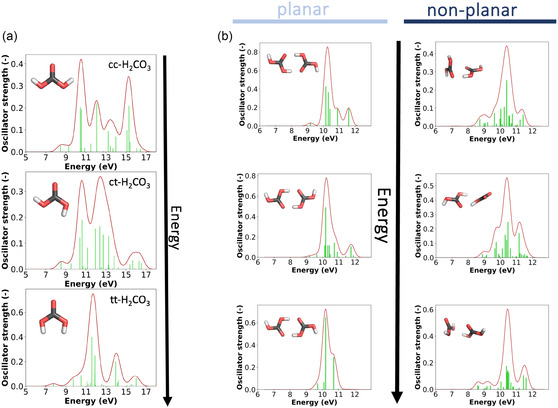
TD‐DFT calculated UV–Vis spectra (CAM‐B3LYP/def2‐SVP) of a) the cc‐H_2_CO_3_, ct‐H_2_CO_3_, and tt‐H_2_CO_3_ **monomers** and b) selected planar and nonplanar H_2_CO_3_ **dimers**, sorted from lowest to highest relative electronic energy. The nonplanar dimers are higher in energy than the planar dimers.

The TD‐DFT calculated UV–Vis spectrum of the **monomer** (cf. **Figure** **4**a) for cc‐H_2_CO_3_, ct‐H_2_CO_3_, and tt‐H_2_CO_3_ spans from 115 nm (8 eV) to 73 nm (17 eV), which exceeds the range covered by our UV experiments. The most intense line at 113 nm (11 eV) in our UV–Vis absorption spectrum compares well to the ionization threshold observed by Kanayama et al.,^[^ ^47^ ^]^ which represents the AIE. While our observed line is not an AIE but an absorption feature, we compare it to the AIE as it is currently the closest available reference for the energetic region. The calculated spectra show additional features: for cc‐H_2_CO_3_, multiple transitions occur at 124 nm (10 eV), 103 nm (12 eV), 89 nm (14 eV), and 83 nm (15 eV) to 77 nm (16 eV). In the case of ct‐H_2_CO_3_, the transitions around 77 nm (16 eV) decrease, while those around 103 nm (12 eV) to 89 nm (14 eV) increase. For tt‐H_2_CO_3_, transitions are predominantly observed around 103 nm (12 eV), with additional features at 89 nm (14 eV) and a peak near 77 nm (16 eV).

For the **dimer**, we found 32 conformers in an energy window of 95 kJ mol^−1^, with the three most stable planar structures being cc‐H_2_CO_3_‐cc‐H_2_CO_3_, ct‐H_2_CO_3_‐ct‐H_2_CO_3_, and tt‐H_2_CO_3_‐tt‐H_2_CO_3_, with relative electronic energies of 0.0, 4.7, and 8.4 kJ mol^−1^. The spectra (cf., Figure 4b, left column) range from 138 nm (9 eV) to 103 nm (12 eV), showing a slight redshift in comparison with the AIE of the monomer. While nonplanar arrangements were also found to be stable structures, these are significantly higher in energy with ≈60 kJ mol^−1^. Although these conformers are hardly populated at atmospheric or even ambient conditions, it is worth noting that nonplanar arrangements result in a redshift of the UV–Vis spectra with new peaks emerging at and below 9 eV (cf., Figure 4b, right column). In addition, many other conformers with the planar arrangement of the two monomer units were found (Figure S4, Supporting Information). These higher energy conformers show transitions at and below 9 eV, indicating symmetry‐lowering and orientations that do not allow for optimal hydrogen bonding also contribute to a redshift. Indeed, when noncovalent interactions are analyzed in these dimers (see Figure S6, Supporting Information), such interactions not only exist in the form of hydrogen bonds but also the form of van der Waals interactions. Considering the dimer conformers shown in Figure 4, the planar ones have each two hydrogen bonds. In contrast, the nonplanar ones have a hydrogen bond and a weak interaction between the two carbonyl (C=O) groups.

#### 2.2.2 Oligomers

For the **trimer**, we found 136 conformers from which the all‐cc‐H_2_CO_3_ conformer is the most stable. Its spectrum ranges from 138 nm (9 eV) to 113 nm (11 eV) (cf., Figure S7, Supporting Information). Exemplary planar and nonplanar trimer structures exhibit a redshift similar to the dimer's. Decreasing planarity and noncovalent intermolecular OH–OH interaction leads to a small redshift for these species.

For the **tetramer**, more than 400 conformers were found. Out of this structural ensemble, we discuss selected low‐energy conformers and conformers that show structural motifs with significant redshift or lack thereof. In the **tetramer**, transitions range from 131 nm (9.5 eV) to 118 nm (10.5 eV) and are further redshifted (see Figure S9, Supporting Information). A transition at 138 nm (9.0 eV) emerges for a structure with noncovalent carbonyl–hydroxyl and hydroxyl–hydroxyl interactions. The size of the redshift depends on the individual orientation of the four monomers toward each other; see also Figure S10 in the Supporting Information, and it does not strictly correlate with energy.

**Figure 5:**
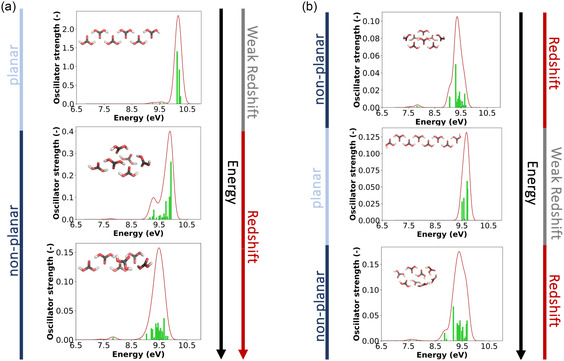
TD‐DFT calculated UV–Vis spectra (CAM‐B3LYP/def2‐SVP) for the lowest energy conformer and selected higher energy conformers of a) the **hexamer** and b) the **octamer**, sorted from low‐to‐high relative electronic energy.

Similar observations were made for the **pentamer** (see Supporting Information) and **hexamer** spectra (**Figure** **5**a), which range from 138 nm (9.0 eV) to 118 nm (10.5 eV) and which are further redshifted. For planar **hexamers**, there are mainly transitions around 124 nm (10.0 eV) to 118 nm (10.5 eV). The number of transitions around 131 nm (9.5 eV) increases for nonplanar species. Furthermore, weak transitions from 165 nm (7.5 eV) to 155 nm (8.0 eV) are observed for the nonplanar conformers with a sheet‐like substructure. With an increasing number of H_2_CO_3_‐monomer units, the energy difference between the planar all‐cc‐H_2_CO_3_ conformer and nonplanar structures decreases, and already for the **hexamers**, it is only 11.5 kJ mol^−1^ in favor of a planar arrangement.

For the **septamer**, the all‐cc‐H_2_CO_3_ conformer is no longer the most stable structure but a nonplanar arrangement of monomer units. In the case of the **septamer**, excitations peak around 131 nm (9.5 eV) (see Figure S15 in the Supporting Information). Interestingly, not only do the nonplanar structures show a significant redshift but also a sheet‐like arrangement of planar H_2_CO_3_ units is found that is redshifted, showing the emergence of low energy excitations at around 161 nm (7.7 eV). This indicates that nonplanarity and noncovalent interactions between two planar sheets can result in redshift as well (compare also later sections).

For the **octamer** (cf., Figure 5b), the number of transitions below 131 nm (9.5 eV) increases for nonplanar clusters, while for the planar all‐cc‐H_2_CO_3_ structure, transitions around 131 nm (9.5 eV) dominate. Notably, this structure is significantly higher in energy, with 37.4 kJ mol^−1^ relative to the most stable one. Similar to the hexamer, there are some weak transitions from 165 nm (7.5 eV) to 155 nm (8.0 eV) for the nonplanar conformers.

We observed that the redshift is correlated with a nonplanar arrangement but not necessarily with the energy of the conformer. For the smaller oligomers, the planarity decreases with increasing conformer energy. In contrast, for the larger **octamer**, the two nonplanar conformers depicted in Figure 5b show a similar redshift. Still, they are significantly different in energy, with the one at the bottom being 44.3 kJ mol^−1^ higher in energy.

For the **16mer**, the **24mer**, and the **48mer**, nonplanar structures dominate with many conformers in a small energy range. For the **48mer**, TD‐DFT calculations must be limited to a few selected species due to the increasing computational cost with system size. From the spectra depicted in the Supporting Information, Figure S18–S23, we observe a further redshift with increasing system size with the main transitions ranging from 177 nm (7.0 eV) to 131 nm (9.5 eV). While we still observe differences in the spectra of the various **16mer** conformers, we see that these differences decrease as we consider the larger **24mer** and **48mer** structures. We speculate that the larger the cluster becomes, the more likely the various substructure motifs that play a role in the redshift are present.

#### 2.2.3 Redshifts from Nonordered Structural Motifs

**Figure 6:**
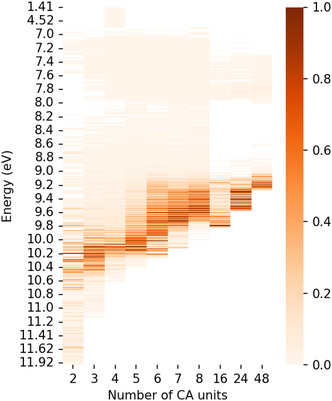
“Redshift” in the calculated UV–Vis spectrum for clusters of up to 48 carbonic acid (CA) units. The TD‐DFT calculated UV–Vis spectra of the respective lowest energy conformer are plotted against cluster size. The color bar shows the normalized UV–Vis intensity within each cluster size.

As outlined, a significant redshift in the calculated UV–Vis spectra becomes apparent with increasing oligomer size. For a systematic comparison, we visualize the spectra for each cluster size for the most stable conformation, starting from the monomer to the **48mer** in **Figure** **6**. In this representation, the UV–Vis intensities are normalized within each cluster and indicated by a color bar. For the dimer, multiple excitations have strong intensities over a wider spectral range. With increasing cluster size, however, quasidegenerate and intense excitations overlap, resulting in a dominant band. Ultimately, these dominant bands redshift toward lower energies upon the increase in cluster size.

With increasing cluster size, the oligomerization leads to various tangled conformers for each oligomer, and the number of conformers drastically increases with the number of H_2_CO_3_ units. The larger the cluster, the more low‐energy conformers emerge, which must be considered in a Boltzmann‐weighting based on the electronic energy of the respective conformers to yield a more accurate spectrum. However, considering the temperature of 5 K, at which amorphous H_2_CO_3_ is studied in our experiments, only the lowest energy conformers contribute. In agreement with the previously discussed results, the Boltzmann‐weighted spectra demonstrate a significant redshift (cf., Figure S24, Supporting Information). However, it is also visible that a significant redshift only occurs once a nonplanar 3D structure becomes the energetically most favorable one, that is, with the **septamer** and larger clusters. Earlier studies found that the linear arrangement is only stable for up to 5 monomer units of H_2_CO_3_, but these differences are likely due to different electronic structure methods.^[^ ^54^ ^]^

Interestingly, Fortenberry and co‐workers assigned the redshift toward 6.5 eV to nonequilibrium structures.^[^ ^60^ ^]^ While it remains to be seen whether such structures exist under experimental conditions, our study of equilibrium structures only (all structures are energy minima) already shows the correct qualitative trend with the emergence of peaks at 7.5 eV. Investigating even larger clusters than the ones shown here would be desirable; however, such calculations become computationally unfeasible. Hence, the band closest to our experimental band at 6.5 eV is at 7.5 eV as observed for the **48mer**. Nevertheless, the present study illustrates the trend, and we expect it to continue for even larger clusters.

Of course, these evaluations assume that our conformer search is complete or at least yields an extensive ensemble capturing all low‐energy conformers. Over the past years, CREST has been established as a conformational sampling tool for various systems. The underlying GFN2‐xTB provides suitable structures as long as no typical transition‐metal complexes are considered. However, we reoptimized the obtained conformers with conventional DFT to increase accuracy. While the conformer search is mostly exhaustive, we observe for selected structures, for example, for the **tetramer** and the **pentamer**, that the most stable planar all cc‐H_2_CO_3_ oligomer is not found but often only a ct‐cc‐cc‐cc or ct‐cc‐cc‐ct **tetramer** species. However, comparing the resulting spectra shows these differences have a minor effect on the spectrum (compare Figure S9, Supporting Information).

The experimental UV–Vis spectrum of isotopically modified H_2_CO_3_ shows a decrease in the line‐broadening with heavier isotopes. However, the peak position remains the same for all isotopologues. This experimentally observed line‐broadening can be partially related to the rovibrational fine structure, which changes with molecular mass. Note that our calculated UV–Vis spectra do not include such isotope effects. We model electronic excitations in the Born–Oppenheimer approximation on each conformer, neglecting any naturally occurring line broadening due to nuclear motions and rovibrational couplings.

To distinguish between size effects and effects due to the absence of long‐range order on the redshift, we also investigated ordered structural motifs cut out of proposed crystal structures without further optimization. For such species, even larger structures consisting of up to 66 monomer units could be considered, as discussed in the following.

#### 2.2.4 Redshift from Ordered Structural Motifs

Additionally, regarding cluster formation, we investigated ordered structural motifs extracted from proposed crystal structures with limited conformational space to distinguish between size effects and effects due to the absence of long‐range order on the redshift. For such species, even larger structures consisting of up to 66 monomer units could be considered, as discussed in the following.

Without an experimentally determined crystal structure of carbonic acid, we relied on computational models proposed by S. Price and co‐workers.^[^ ^34^ ^]^ From their computationally generated structures, we selected four that are energetically favored and differ significantly in monomers’ alignment and spatial arrangement. Each structure provides a distinct configuration, enabling a comparative analysis of their UV–Vis properties in response to monomer arrangement.

**Figure 7:**
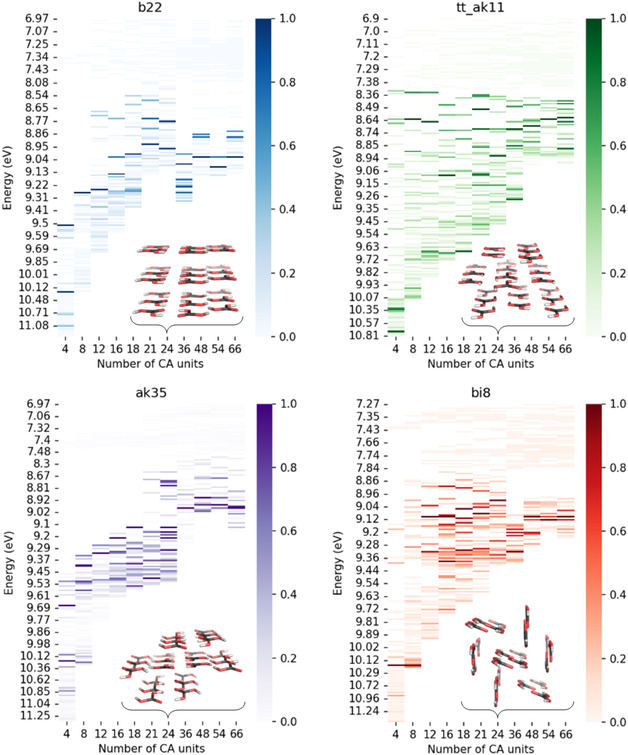
“Redshift” in the calculated UV–Vis spectrum for ordered clusters of up to 66 carbonic acid (CA) units for different computationally proposed crystal structures, including planar structures **b22** and **tt‐ak11** and slightly nonplanar **ak35** and strictly nonplanar **bi8** structural motifs. The TD‐DFT calculated UV–Vis spectra are plotted against cluster size. The color bar shows the normalized UV–Vis intensity. Each panel shows the 24 mer as a representative for the respective structural motifs.

**Figure** **7** includes depictions of the considered structures, with the **24mer** as representatives. The structure denoted as **b22** features parallel planes composed of ct‐H_2_CO_3_ monomers, with each successive layer exhibiting a slight lateral displacement. The **tt‐ak11** structure also exhibits parallel planes but consists of cc‐H_2_CO_3_ monomers, with layers displaced relative to one another. The **ak35** structure departs from planarity, displaying slightly nonplanar layers of ct‐H_2_CO_3_ monomers. Finally, **bi8** forms chains of cc‐H_2_CO_3_ monomers that, within a given layer, are nearly coplanar but are slightly tilted, with each successive layer showing an alternating tilt angle; for more structural details, see Figure S25–S28, Supporting Information.

We created smaller crystal structure slabs starting from a **66mer** by removing carbonic acid monomer units, resulting in multiple possible constitutional isomers for each *n*‐mer. As shown in Figure 7, an observable redshift emerges in all structures with increasing H_2_CO_3_ units. We consistently cut the hypothetical crystal structures, but this approach may have introduced spectral discontinuities (e.g., **24mer** to **36mer** in **b22** and **ak35**). Although such truncations cause dark states in specific regions, the overall redshift trend compared to the monomer remains clear.

Specifically, for the planar structures **b22** and **tt‐ak11**, the redshifts extend below 9 eV, reflecting a consistent shift in electronic transitions with cluster size (cf., Figure 7); for individual structures and spectra, see Figure S25 and S26, Supporting Information. A similar redshift is observed in the slightly nonplanar **ak35** and **bi8** structure, with the primary absorption intensities redshifted toward 8–9 eV (cf., Figure 7, individual spectra are displayed in Figure S27 and S28, Supporting Information). As cluster size increases, the **bi8** structure also shows a progressive redshift. However, it additionally reveals low‐intensity peaks between 7 and 8 eV, which are absent in the strictly planar **b22** and **tt‐ak11** structures. This trend is consistent with our observations for the nonordered, nonplanar structural motifs in the above calculations and best fits the experimentally observed redshift. Visualizing noncovalent interactions (Figure S29, Supporting Information) reveals that weak van der Waals interactions between chains and sheets occur in addition to hydrogen bonds. These interactions are believed to contribute to the redshift, as discussed for the dimer before.

In addition, we analyzed a proposed high‐pressure structure of D_2_CO_3_ to complement this set, which we modified to H_2_CO_3_ and reoptimized, in particular the hydrogen positions, for our computational study. This high‐pressure configuration exhibits a qualitatively similar redshift with cluster size (cf., Figure S30, Supporting Information); however, the absolute wavelengths of the transitions in this structure differ notably from those observed in the four aforementioned carbonic acid structures. The variance in wavelength is attributed to the high pressure and the high‐energy structure unique to the high‐pressure polymorph of the D_2_CO_3_ reference data.

In summary, we observe a redshift with increasing cluster size across all planar and nonplanar structures. Nevertheless, additional low‐energy peaks emerge for the nonplanar configurations, including **ak35** and **bi8**, as cluster size increases, features absent in completely planar structures such as **b22** and **tt‐ak11**. This difference in spectral behavior highlights the influence of nonplanarity and monomer alignment on the electronic properties of carbonic acid clusters.

## 3 Conclusion

We present new laboratory spectroscopy data of solid H_2_CO_3_ obtained from ice mixtures and utilized TD‐DFT to rationalize the redshift in the UV–Vis spectra by studying H_2_CO_3_ oligomers of increasing size. IR spectroscopy confirms that the H_2_CO_3_ sample is a mixture of amorphous and crystalline solid phases. To explain the redshift in the UV–Vis region, that is the shift of the maximum absorption band with cluster size compared to the monomer, we conducted a systematic computational study, starting from the H_2_CO_3_ monomer and extending to cluster arrangements containing up to 66 H_2_CO_3_ monomer units. In contrast to previous studies, we systematically increased the cluster size to study nonordered structural motifs and investigated slabs of proposed ambient‐pressure H_2_CO_3_ crystal structures. By accounting for conformational diversity through extensive sampling and Boltzmann weighting, we ensured that all presented structures are energy minima. While oligomerization has previously been shown to cause a redshift in UV–Vis spectra,^[^ ^6^, ^22^, ^59^, ^61^ ^]^ our study provides further insight into the correlation of structural motifs and spectral shift.

Our experimental UV–Vis spectrum of solid H_2_CO_3_ shows a broad absorption band at 194 nm (6 eV). Relative to the gas‐phase AIE of the monomer reported at ≈113 nm (6 eV) by Kanayama et al.,^[^ ^47^ ^]^ our observed absorption is shifted by ≈80 nm, which corresponds toa shift of ≈5 eV on the energy scale. Similarily, with respect to the AIE of the monomer, a second shift of ≈25 nm (or ≈2 eV on the energy scal) can be defined on the UV–Vis spectra by Ioppolo et al.,^[^ ^22^ ^]^ who report a second band at 138 nm (9 eV). Although direct assignment of the excited‐state character is beyond the scope of this study, both redshifts can be rationalized based on our calculated structural models.

The redshift of ≈2 eV already appears in small oligomers, where the dominant peak centers at ≈9 eV for the largest investigated clusters (**48mer**). This redshift results from 1) increasing cluster size and 2) a nonplanar arrangement of H_2_CO_3_ monomer units, and 3) noncovalent interactions within and between H_2_CO_3_ monomers, as visualized by noncovalent interaction plots (Figure S6, Supporting Information).

A more pronounced redshift of ≈5 eV is indicated in our calculations, with a prominent peak emerging near ≈7.5 eV for larger clusters, suggesting that cluster growth and intermolecular interactions significantly impact the absorption profile. Wallace et al.^[^ ^60^ ^]^ attributed such shifts to nonequilibrium structures, that is, nonenergy‐minimum conformations of τ(OCOO) dihedral angles (“ribbon structures”); however, the stability of these conformations under experimental conditions remains unclear. In contrast, our findings demonstrate that even energy‐minimum conformations can exhibit substantial redshifts.

Additionally, our investigations on slabs of proposed H_2_CO_3_ crystal structures confirm that redshifts occur even in regular arrangements. These shifts can be attributed to noncovalent interactions between sheets or chains of planar H_2_CO_3_ units present in all proposed structures (as visualized in the noncovalent interaction plots Figure S29, Supporting Information) and decreasing planarity, observed, for example, in the hypothetical structure **bi8**. We suggest that these factors contribute further to the observed redshift.

It is important to note that our discussion of UV–Vis redshifts in referece to the adiabatic ionization energy of the H_2_CO_3_ monomer, reported by Kanayama et al.,^[^ ^47^ ^]^ is an approximation. The TD‐DFT calculated UV–Vis spectra correspond to neutra‐neutral exciations, not direct ionaization. Although our TD‐DFT results capture trends in spectral shifts due to clustering and structural disorder, they cannot directly yield AIEs. However, the proximity of computed excitation energies to the ionization threshold suggests that Rydberg excitations may be present and should be considered in interpreting VUV absorption features. This distinction is crucial, particularly when comparing UV–Vis spectra to photoelectron spectroscopy data that probe ionized states.

In summary, fully capturing the ≈5 eV redshift would require significantly larger computational models, which remain beyond current capabilities. In addition, experimental data of solid H_2_CO_3_ in the VUV region is still sparse, and the community would benefit from further studies. Nevertheless, our analysis reveals systematic trends in UV–Vis spectral shifts with increasing H_2_CO_3_ cluster size. Without comprehensive laboratory spectra of H_2_CO_3_, our findings provide valuable computational reference data to support future laboratory studies and astrochemical observations. By bridging this experimental gap, our work enhances our understanding of spectrum‐to‐structure relationships. Ultimately, laboratory UV–Vis spectra of H_2_CO_3_ oligomers would be invaluable for validating computational models and refining these relationships, which are essential for interpreting water–carbon dioxide ices in the interstellar medium. After this manscript was accepted for publication a new high pressure, high temperature crystal structure of H_2_CO_3_ was published, which would be worth investigating in the future.^[^ ^69^ ^]^

## 4 Experimental Section

4.1

4.1.1

##### Experiment

H_2_CO_3_ was prepared from irradiated H_2_O and CO_2_ ice mixtures in an ultra‐high vacuum experimental setup.^[^ ^70^, ^71^ ^]^ The main chamber has a base pressure of 10^−11^ Torr obtained using oil‐free magnetically suspended turbomolecular pumps and dry scroll backing pumps. A closed‐cycle helium (Sumitomo, RDK‐415 E) cryostat cools a rhodium‐coated silver mirror (1.5 × 1.2 cm) to a low temperature of 5.0 ± 0.1 K and is freely rotatable within the horizontal plane of the chamber. Rhodium coating was used to increase the overall reflectance in the UV electromagnetic region and to avoid the optical absorption of pure silver at around 320 nm.^[^ ^72^ ^]^ Upon reaching the temperature of 5.0 K, the gas phase water (H_2_O, HPLC, Fisher Chemical) and carbon dioxide (CO_2_, 99.999%, Airgas) were then deposited through two glass capillaries with a background pressure reading in the main chamber of 4 × 10^−8^ Torr for ≈8 min to yield an 850 ± 50 nm ice sample. The thickness of the sample was determined by in situ monitoring the ice deposition with a helium‐neon laser (CVI Melles‐Griot, 25‐LHP‐230, 632.8 nm) at a 4° angle of incidence and measuring the interference induced by the deposited thin film ices.^[^ ^73^, ^74^ ^]^ The refractive indices (n) of water and carbon dioxide ices, necessary to determine thickness from interferometric measurements, were approximated to be 1.27 ± 0.02. This value was obtained by averaging the refractive indices of the two components used in the literature: water of 1.27 ± 0.02 and carbon dioxide of 1.27 ± 0.02.^[^ ^75^ ^]^ The ratio of water–carbon dioxide ice mixture was determined to be (1.1 ± 0.1):1 by integration and subsequent calculation of the column density using a modified Beers–Lambert law^[^ ^76^, ^77^ ^]^ of the CO_2_ at 3703 cm^−1^ with the integral absorption coefficient of 1.8 × 10^−18^ cm molecule^−1^ and integration of the H_2_O band at 1643 cm^−1^ using an integral absorption coefficient value of 9.0 × 10^−^^18^ cm molecule^−1^. The ice samples were then irradiated with 5 keV electrons isothermally at 5.0 ± 0.1 K over a square area of 0.9 ± 0.1 cm^2^ and at an angle of 70° relative to the surface normal. The emissions current was measured before and after irradiation utilizing a Faraday cup (Kimball Physics, FC‐71) mounted on an ultrahigh vacuum compatible translation state. The total dose deposited into the ice sample was then determined utilizing a Monte Carlo electron simulation (CASINO) package.^[^ ^78^ ^]^ The total energy deposited into the ice mixtures was determined to be 47.6 ± 5.0 eV amu^−1^. After irradiation, the ices were warmed up to 210 K, kept for 30 min, and then cooled down to 5 K. The heating process is to desorb the reactants water and carbon dioxide or possible products of formaldehyde (H_2_CO_3_), leaving pure products of carbonic acid.^[^ ^13^ ^]^ A Fourier Infrared Transform Spectrometer (Nicolet 6700) monitored the samples throughout the experiment with an IR spectrum collected every two minutes (110 scans per minute) in the range of 6000–400 cm^−1^ at a resolution of 4 cm^−1^. The column density of H_2_CO_3_ at 5 K was calculated to be 1.97 × 10^16^ molecule cm^−2^ using the absorption band area at 1508 cm^−1^, and the corresponding integral absorption coefficient value of 6.5 × 10^−17^ cm molecule^−1^.^[^ ^15^ ^]^ The thickness of carbonic acid at 5 K was estimated to be about 20 ± 2 nm by using its column density and the density of 0.99 g cm^−3^, which is obtained by averaging that of water and carbon dioxide for 0.87 ± 0.03 and 1.11 ± 0.03 g cm^−3^.^[^ ^75^ ^]^ A quadrupole mass spectrometer (Extrel, Model 5221) operating in residual‐gas analyzer mode in the mass range of 1–200 amu with electron impact ionization energy of 70 eV allows detecting species in the gas phase throughout the experiment. A modified UV–Vis spectrophotometer (Nicolet Evolution 300 with pulsed xenon lamps) aided in the recording of absorption spectra in the range of 190–1100 nm with a resolution of 4 nm at a scan speed of 120 nm minute^−1^. Here, light from the spectrophotometer was directed onto the cryogenic ice via an enhanced aluminum‐coated mirror (ThorLabs, PF10‐03‐F01) through UHV‐compatible MgF_2_ 35 CF viewports (Kurt J. Lesker, VPZL‐275UM) while being focused by a cylindrical lens (CVI Melles‐Griot SCX‐25.4‐381.4‐UV) to compensate for the divergence in the vertical direction, upon which the beam was reflected off the rhodium coated silver at an angle of 30° onto UV enhanced parabolic mirror (Edmund Optics, 63‐180), which then focused the light onto the original detector now placed into a black N_2_ purged box that effectively shielded any ambient lights.

##### Computational Methodology

The minimum energy structures of the **monomer** were created with Chemcraft^[^ ^79^ ^]^ and optimized using DFT with the hybrid B3LYP density functional^[^ ^80^, ^81^, ^82^, ^83^ ^]^ in combination with the Karlsruhe def2‐SVP basis set^[^ ^84^ ^]^ and empirical dispersion correction of the D4 type.^[^ ^85^ ^]^ For benchmarking, selected calculations were also performed with the def2‐TZVP basis set.^[^ ^84^ ^]^ The B3LYP density functional has previously been used to study H_2_CO_3_ conformers, and we adhere to this choice to facilitate comparison of our results to previous works.^[^ ^6^, ^60^ ^]^ From these optimized monomer structures, one initial conformer for each oligomer (dimer, trimer, etc.) was created in Chemcraft^[^ ^79^ ^]^ and preoptimized with GFN2‐xTB.^[^ ^86^ ^]^ For each oligomer, a conformation sampling was performed with CREST^[^ ^68^ ^]^ with a threshold for the root mean square deviation between two conformers of 0.1 Å and an energy threshold of 0.1 kcal mol^−1^. Structures within an energy threshold of 15 kcal mol^−1^ were considered for the final CREST conformer ensemble. All CREST‐identified conformers were reoptimized with DFT using B3LYP/def2‐SVP/D4. A detailed comparison of GFN2‐xTB and DFT optimization schemes can be found in the Supporting Information. However, optimizing all conformers with DFT was only feasible up to the **octamer** as the number of structures quickly reached several hundreds of conformers. For the larger systems (**16mer**, **24mer**, and **48mer**), only 10 manually selected conformers were reoptimized with DFT to manage the increasing computation times.

TD‐DFT calculations were performed with the Tamm–Dancoff approximation to calculate the excited states. As B3LYP could be problematic for UV–Vis spectra calculation, we used the range‐separated CAM‐B3LYP density functional^[^ ^87^ ^]^ in conjunction with def2‐SVP instead. To speed up the calculation, the RIJCOSX approximation^[^ ^88^ ^]^ was used together with a tight SCF convergence criterion. All DFT and TD‐DFT calculations were performed using ORCA.^[^ ^89^ ^]^ The resulting UV–Vis spectra were visualized using a Python script. A Boltzmann weighting was applied for each cluster size to account for the structural ensemble at a given temperature (5 K) to yield the final UV–Vis spectra (for details, see Supporting Information). Clusters beyond 24 monomers resulted in hundreds of CREST conformers, which would require reoptimization with DFT due to the known discrepancy between GFN2‐xTB and DFT results. However, we refrained from further refinement of the ensemble with DFT due to prohibitive computational costs. Absorption spectra were obtained by plotting the excitation energies against the oscillator strength and applying a Gaussian line‐broadening.

We also investigated slabs of proposed carbonic acid crystal structures and the recently reported D_2_CO_3_ species to compare the effect of increasing size on the UV–Vis spectra.^[^ ^34^, ^62^ ^]^ Clusters of 66, 48, 36, 24, 21, 18, 16, 12, 8, and 4 monomers were generated from the available crystal structure data to prepare the slabs. These slabs, derived from the proposed crystal structures of Winkler et al., were directly subjected to TD‐DFT studies using the same settings as the amorphous clusters without additional optimization. As the experimental structure was a D_2_CO_3_, a re‐optimization with H instead of D was performed on a 120 monomer slab before UV–Vis calculations. The resulting UV–Vis spectra were visualized in Chemcraft, and a Gaussian line‐broadening was applied. All structures were visualized with PyMOL.^[^ ^90^ ^]^

Noncovalent interactions were calculated and visualized using an isosurface of the reduced density gradient proposed by Johnson et al.^[^ ^91^ ^]^ On these isosurface patches the 2nd eigenvalue of the Laplacian of the density is plotted, which indicates strongly attractive interactions, such as hydrogen bonds, weakly attractive interactions, such as van der Waals interactions, and repulsive interactions, such as steric repulsion. Calculations were performed with the NCIPlot program,^[^ ^92^ ^]^ which requires a wfn file, which, in turn, was generated using Turbomole^[^ ^93^ ^]^ with the same density functional and basis set as the ORCA calculations. Although the two programs, even when using the same methodology, never yield the exact same total energies, this is of no concern. Only the density is analyzed here, which is largely insensitive to the exact electronic structure method used to calculate it. Noncovalent interaction plots were visualized with VMD.^[^ ^94^ ^]^

## Conflict of Interest

The authors declare no conflict of interest.

## Supporting information

Supplementary Material

## Data Availability

The data that support the findings of this study (cartesian coordinates of all investigated conformers) are available from https://doi.org/10.5281/zenodo.15053061.
