# Supplementary material for: UV–Vis Spectra of Carbonic Acid: Rationalizing Experimental Redshifts between Monomer and Bulk based on (H2CO3)n Calculations
Source: Chemphyschem. 2025 Aug 4;26(18):e202500282. doi: 10.1002/cphc.202500282 (PMC12453297; doi:10.1002/cphc.202500282)
Supplement: Supplementary file 1 — Supplementary Material [file CPHC-26-e202500282-s001.pdf]

UV-VIS spectra of carbonic acid: redshifts from the single molecule to the bulk - Supplementary Information

Contents

1 Non-ordered structural motifs **2**

1.1 Boltzmann weighting of spectra 2

1.2 Performance of GFN2-xTB 2

1.3 Results for *N*mers 4

1.3.1 Monomer 4

1.3.2 Dimer 4

1.3.3 Trimer 6

1.3.4 Tetramer 7

1.3.5 Pentamer 10

1.3.6 Hexamer 11

1.3.7 Septamer 12

1.3.8 Octamer 14

1.3.9 Larger clusters (16mer/ 24mer/ 48mer) 14

1.3.10 16mer 15

1.3.11 24mer 16

1.3.12 48mer 18

1.3.13 Comparison of redshift 19

2 Ordered structural motifs **20**

1 Non-ordered structural motifs

The methodology to calculate the Boltzmann averaged UV-vis spectra and a comparison of the performance of GFN2-xTB and DFT is given before detailed results are presented.

1.1 Boltzmann weighting of spectra

To account for the conformational diversity, we performed a Boltzmann weighting for each cluster size to get an overall spectrum for each cluster. The following equation 1 was used to calculate the Boltzmann weights (ω) for each conformer at 5 K. A cutoff value of the Boltzmann weight was set at 1% since conformers with higher energies play an insignificant role in the overall spectrum.

$$\omega = e^{\frac{-E_i}{k \cdot T}} \tag{1}$$

Boltzmann weighting of the conformers was applied for each cluster size to compute the overall UV-vis spectra.

1.2 Performance of GFN2-xTB

As is well known that the performance of GFN2-xTB critically depends on the system studied, a careful evaluation of its performance against full DFT is necessary. To gauge the precision in an ensemble of structures, we studied the impact of the electronic structure method at the example of the H_2CO_3 **dimer**, for which the CREST search identified 32 conformers with the settings detailed in the Computational methodology section. These conformers were reoptimized with B3LYP/def2-SVP/D4 or B3LYP/def2-TZVP/D4, and the calculated relative DFT energies were plotted against the relative xTB energies. As evident from Fig. 1, both comparisons yield similar results, which is further corroborated by the performed linear regression. While the results indicate that for the majority of the structures xTB and DFT results agree well, there are a few notable exceptions: For two structures, xTB determined a relative energy between 55 and 60 kJ/mol, while DFT predicted them to be very stable with energies between 0 and 5 kJ/mol (cf. Fig. 1). Due to these discrepancy, we considered a reoptimization with DFT necessary.

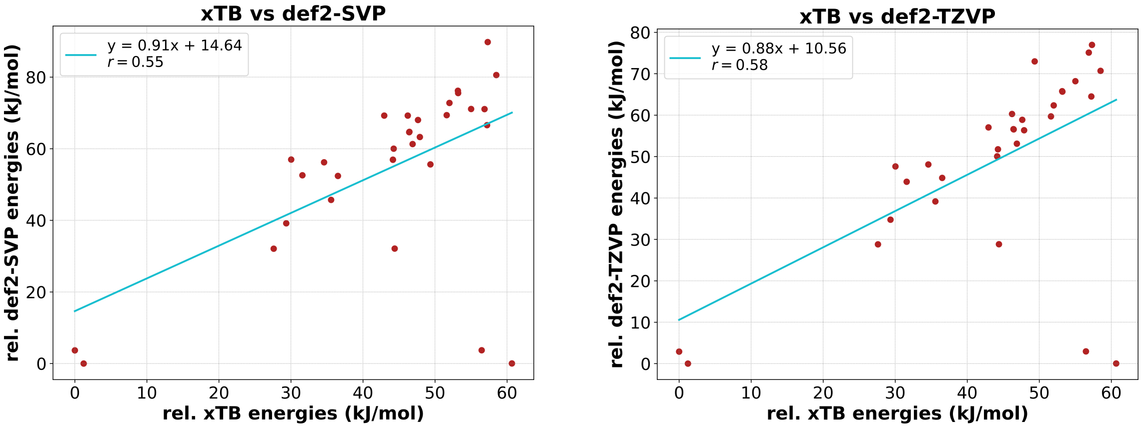

Figure 1: Relative xTB energies (kJ/mol) of the **dimer** conformers plotted against relative DFT energies (kJ/mol) (left: B3LYP/def2-SVP/D4, right: B3LYP/def2-TZVP/D4)

To assess the effect of the basis set, we plotted the def2-SVP optimized structures against the def2-TZVP (cf. Fig. 2 (left)) as well as the def2-TZVP single point energies on the def2-SVP optimized structures against the def2-TZVP optimised conformers (cf. Fig. 2 (right). In both cases, an excellent correlation can be observed. As the def2-TZVP single points on the def2-TZVP structures do not increase the correlation much, we considered the def2-SVP basis set sufficient and a good compromise between accuracy and computational calculation time.

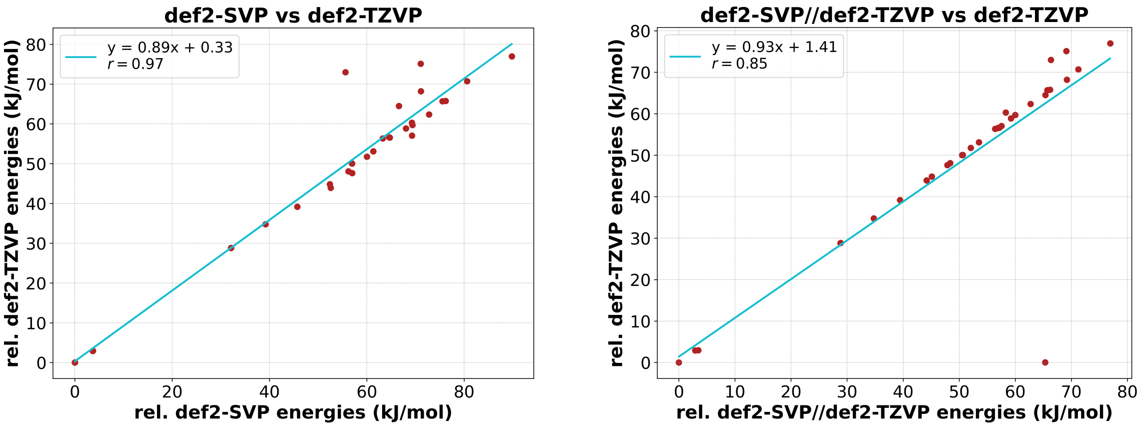

Figure 2: Comparison of calculated DFT energies (kJ/mol) of the **dimer** conformers with different basis sets. Left: relative energies (B3LYP/def2-SVP) against relative energies (B3LYP/def2-TZVP). Right: relative energies (B3LYP/def2-TZVP) against relative energies (B3LYP/def2-SVP//B3LYP/def2-TZVP).

1.3 Results for *N*mers

In the following detailed and additional results for all calculated *N*mers with $N = 1, 2, ...48$ are given.

1.3.1 Monomer

The Boltzmann weighed UV-vis spectra of the **monomers** at 5 K is shown in Fig. 3, only the lowest energy cc-H_2CO_3 conformer contributes to the overall spectrum.

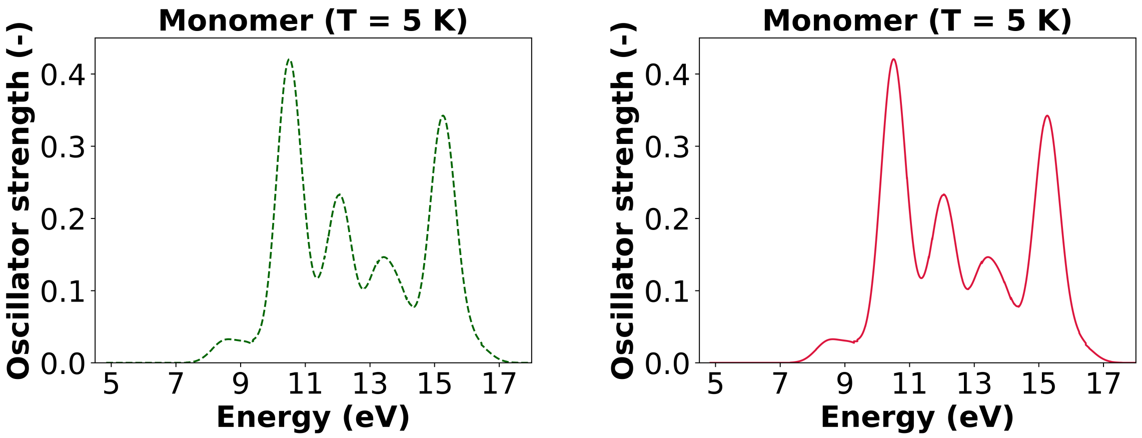

Figure 3: Boltzmann weighting of the **monomer** at 5 K. Left: UV-vis spectrum of each conformer multiplied by the corresponding Boltzmann weight ($\omega > 1\%$). Right: Resulting Boltzmann weighted spectrum.

1.3.2 Dimer

The conformational search with CREST calculated 32 different minima structures. While the most stable planar dimers do not show a redshift and nonplanar structures a strong one, further analysis of additional higher energy planar structures revealed that also here a redshift is observed (see 4). Characteristic of these conformers is are OH-carbonyl and OH-OH noncovalent interactions. Identifying planar structures showing redhifted UV-Vuis spectra is in contrast to previous observations by Fortenberry who attributed the redshift to the nonplanarity.

The Boltzmann weighted UV-vis spectra of the **dimers** are displayed in Fig. 5. Three conformers contributed to Boltzmann weighted UV-vis spectra.

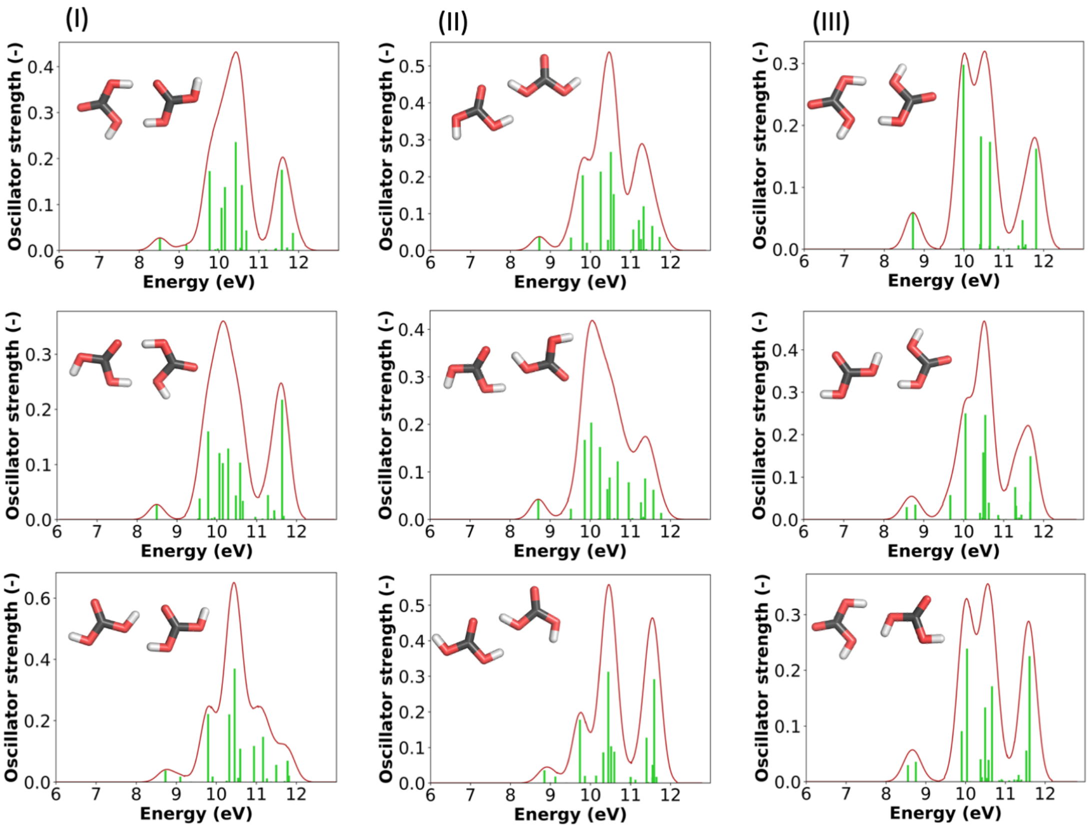

Figure 4: UV-vis spectra of additional planar **dimer** structures. The relative energy increases from top to bottom and from left to right.

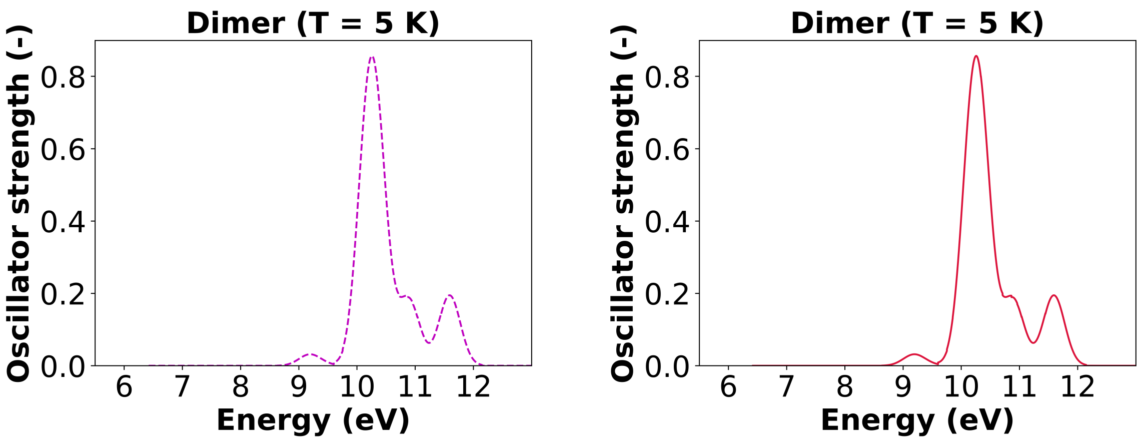

Figure 5: Boltzmann weighting of the **dimer** at 5 K. Left: UV-vis spectrum of each conformer multiplied by the corresponding Boltzmann weight ($\omega > 1\%$). Right: Sum of Boltzmann spectra.

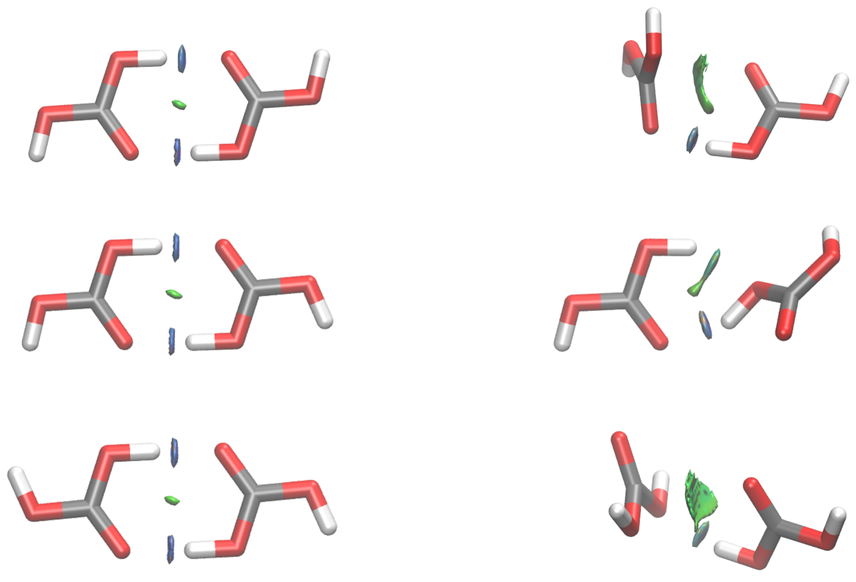

Figure 6: Noncovalent interactions for the dimer structures displayed in Figure 4b in the main manuscript. An isosurface of the reduced electron density gradient of 0.05 a.u. is displayed on which the 2nd eigenvalue of the Laplacian is plotted, which can be used to distinguish strongly attractive noncovalent interactions (depicted in blue), weak, van der Waals like interactions (depicted in green) and repulsive interactions, as arising from steric hindrance (not visible here, but usually depicted in red).

From Figure 6 it is evident that strong attractive hydrogen bonding interactions exist between hydroxyl and carbonyl groups as indicated by blue patches but that also weak van der Waals like interaction are present between for example carbonyl oxygen and carbonyl carbons as indicated by the green patches.

1.3.3 Trimer

The conformational search with CREST yielded 136 conformations for the **trimer**. The lowest energy conformer as well as two additional planar and non-planar structures are depicted in Fig. 7. While the all cc-H_2CO_3 does not show a visible redshift compared to the **dimer**, the a redshift is not observed, it is visible for the higher energy non-planar and planar structure. Again the in both structures, carbonly-OH and OH-OH interactions are visible.

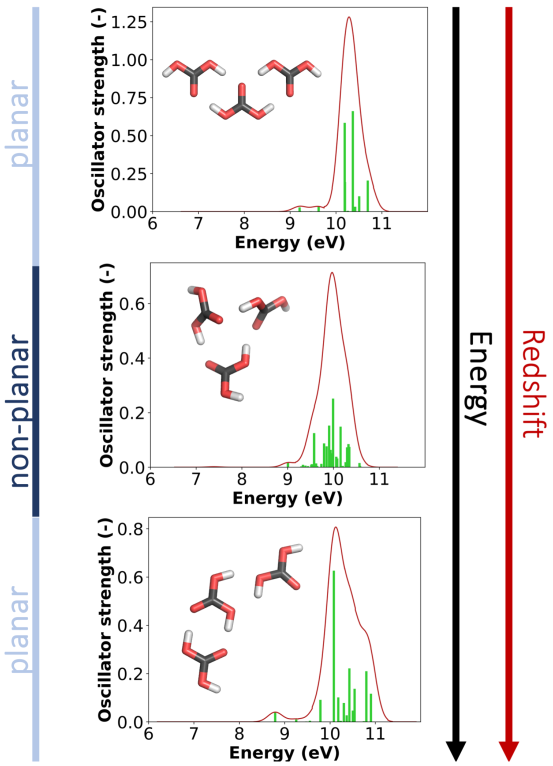

Figure 7: Selected **trimer** conformers and their corresponding UV-vis spectra.

The Boltzmann weighted spectra (see Fig. 8) showed that at 5 K only the all cc-H_2CO_3 conformer contributed to the spectrum.

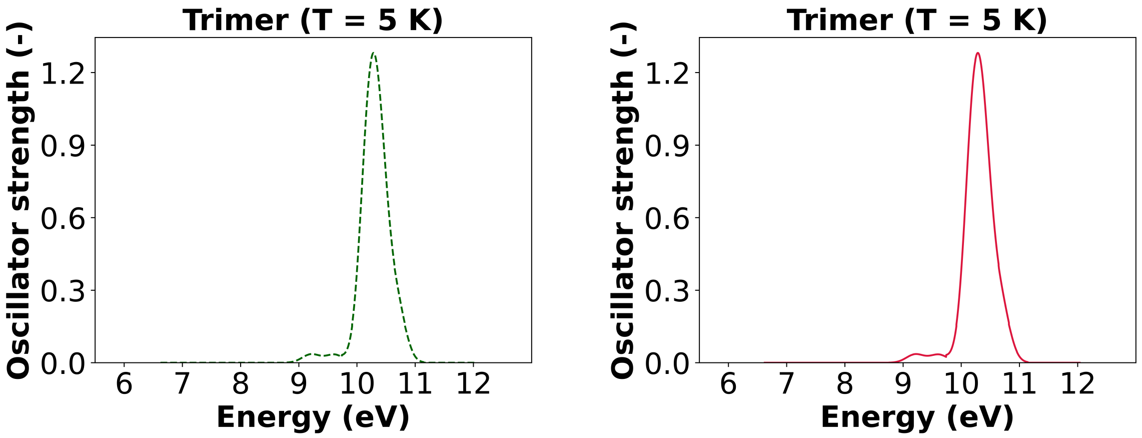

Figure 8: Boltzmann weighting of the **trimer** at 5 K. Left: UV-vis spectrum of each conformer multiplied by the corresponding Boltzmann weight ($\omega > 1\%$). Right: Resulting Boltzmann weighted spectrum.

1.3.4 Tetramer

The conformational search with CREST calculated 409 minima structures. As seen in Fig. 9, the two planar structures cc-cc-cc-ct-H_2CO_3 and the ct-cc-cc-ct-H_2CO_3 showed

very similar UV-vis spectra, depsite an energy difference of 5 kJ/mol. Compared to the cc-cc-cc trimer, a very small redshift is visible. In agreement with previous results, this shift is more pronounced for non-planar structures.

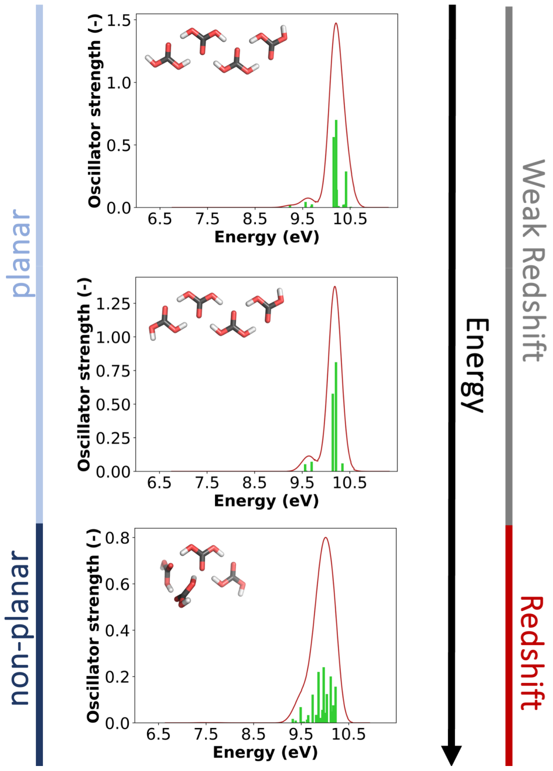

Figure 9: Selected **tetramer** conformers and their corresponding UV-vis spectra showing a redshift with decreasing planarity.

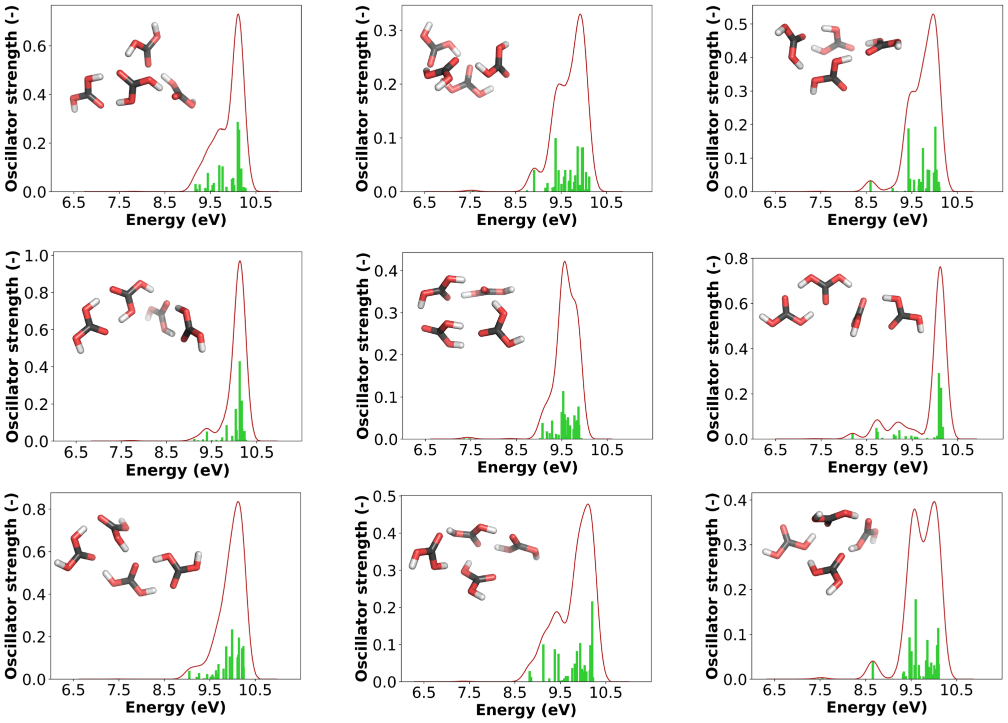

Figure 10: Additional non-planar **tetramer** conformers and their corresponding UV-vis spectra.

From the Fig. 10 it is visible that the redshift depends on the specific non-planar orientations of the monomer units towards each other, while certain orientations resulted in the emergence of lower peaks at 8.5 eV. Although the relative energies of these clusters are somewhat higher compared to the global minimum, it is not only possible but also likely that such motifs occur in an amorphous solid.

For the Boltzmann weighting at 5 K, only the planar lowest energy conformer was included since all other conformers have a weight of less than 1% (cf. Fig. 11).

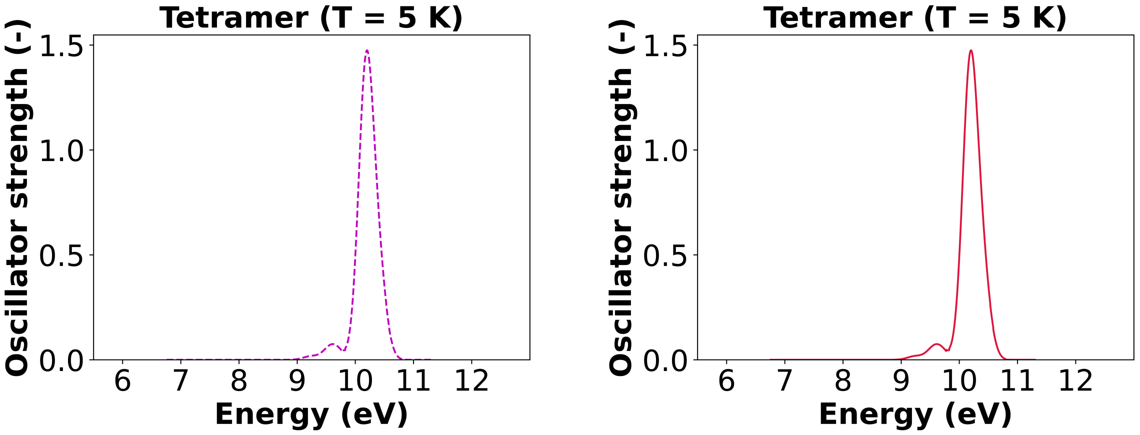

Figure 11: Boltzmann weighting of the **4mer** at 5 K. Left: UV-vis spectrum of each conformer multiplied by the corresponding Boltzmann weight ($\omega > 1\%$). Right: Resulting Boltzmann weighted spectrum.

1.3.5 Pentamer

The conformational search with CREST calculated 743 minima structures for the **pentamer**; however, only 640 structures were re-optimized with DFT as some showed dissociated monomer units and to reduce the calculation time. For the all planar conformation, the redshift compared to the **tetramer** is hardly visible and only visible for non-planar conformations, see Fig. 12. For the Boltzmann weighting at 5 K, only the conformer with the lowest energy contributed. The main peak is at approximately 10.2 eV, slightly shifted compare to the **dimer** and **trimer**.

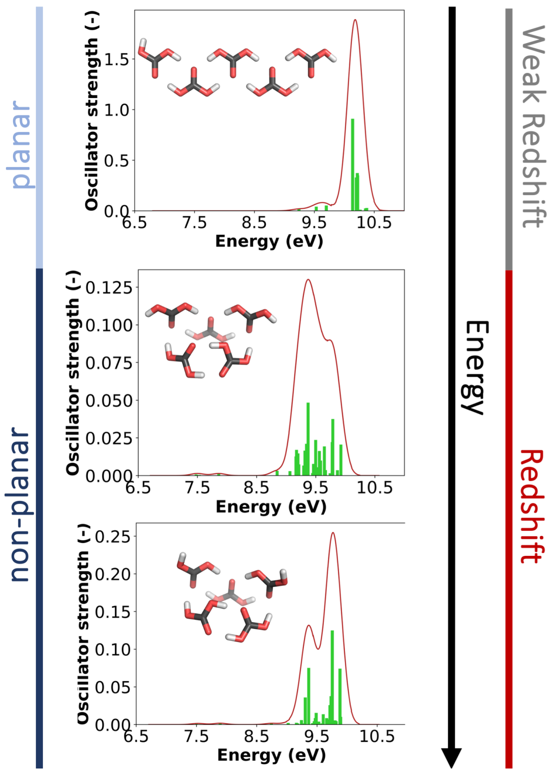

Figure 12: Selected **pentamer** conformers and their corresponding UV-vis spectra showing a redshift with decreasing planarity.

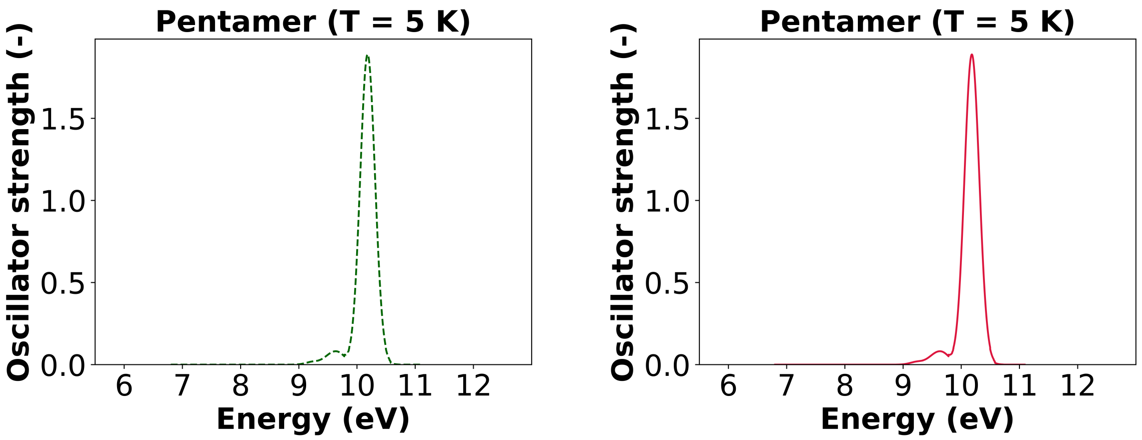

Figure 13: Boltzmann weighting of the **5mer** at 5 K. Left: UV-vis spectrum of each conformer multiplied by the corresponding Boltzmann weight ($\omega > 1\%$). Right: Resulting Boltzmann weighted spectrum.

1.3.6 Hexamer

For the **hexamer** cluster, 675 minima structures were identified using CREST, but only 640 structures were reoptimized to reduce calculation time. As seen in Fig. 14, three conformers were included for calculating the Boltzmann weighted spectra.

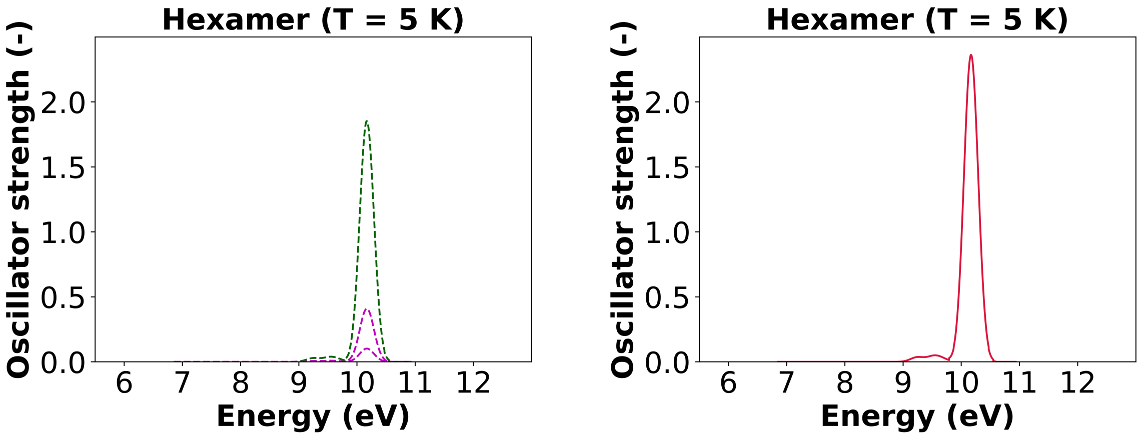

Figure 14: Boltzmann weighting of the **hexmer** at 5 K. Left: UV-vis spectrum of each conformer multiplied by the corresponding Boltzmann weight ($\omega > 1\%$). Right: Resulting Boltzmann weighted spectrum.

1.3.7 Septamer

For the **septamer** cluster, CREST identified 694 energetic minima structures, but a cutoff was set again at 640 to reduce calculation time. A significant difference can be seen for this cluster size compared to the smaller systems: The structure with the lowest energy is non-planar, as visible in Fig. 15.

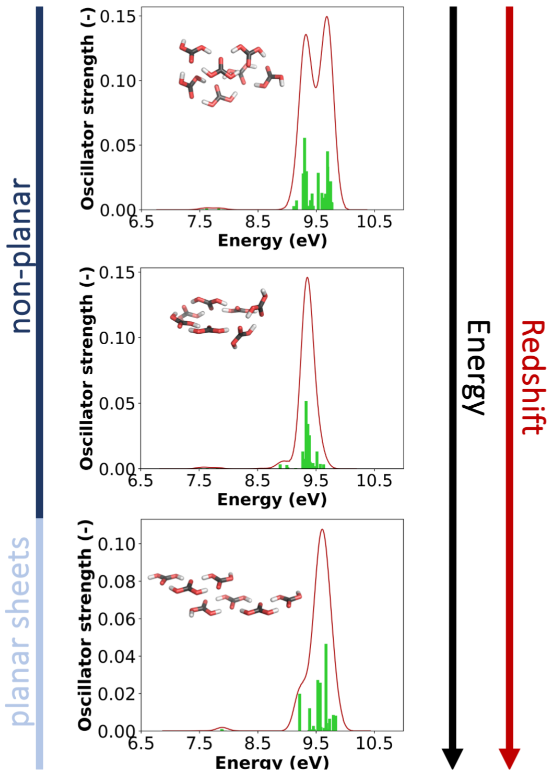

Figure 15: Selected **septamer** conformers and their corresponding UV-vis spectra showing a redshift with decreasing planarity.

Fig. 16 shows that five conformers contribute to the Boltzmann weighting, which indicates that - as for the **septamer** system - many low energy conformations emerge. However, one structure still dominates the spectra.

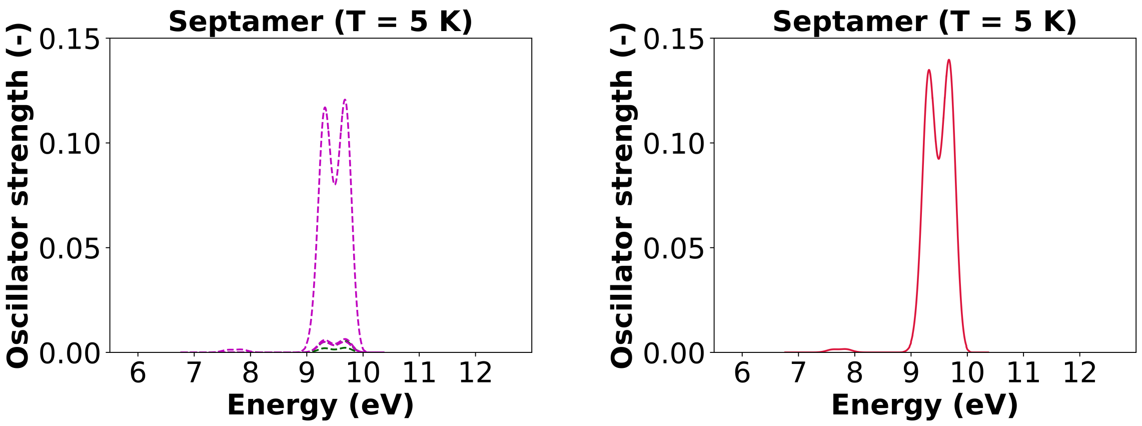

Figure 16: Boltzmann weighting of the **7mer** at 5 K. Left: UV-vis spectrum of each conformer multiplied by the corresponding Boltzmann weight ($\omega > 1\%$). Right: Resulting Boltzmann weighted spectrum.

1.3.8 Octamer

CREST determined 872 minima conformations for the **octamer** cluster. All 872 structures were optimized with DFT (exception was conformer 525, because ORCA was unable to optimize the structure). As the **octamer** calculations were performed before the **pentamer** to **septamer**, all structures were optimized here. For larger cluster sizes, the higher energy CREST conformations did not converge to any low energy DFT conformations, and hence, did not add much additional insight. The results for the **octamer** system are similar to those from the **septamer**: The structure with the lowest energy is non-planer (see main manuscript). Again, a redshift can be observed between planar and non-planar structures. Furthermore, the main peak for the planar systems is located at approx. 9.6 eV indicates a redshift with increasing cluster size (e.g., the main peak position of a planar **5mer**: $E = 10.2\ eV$). The Boltzmann weighting at 5 K of the **octamer**s is shown in Fig. 17, where four conformers contribute.

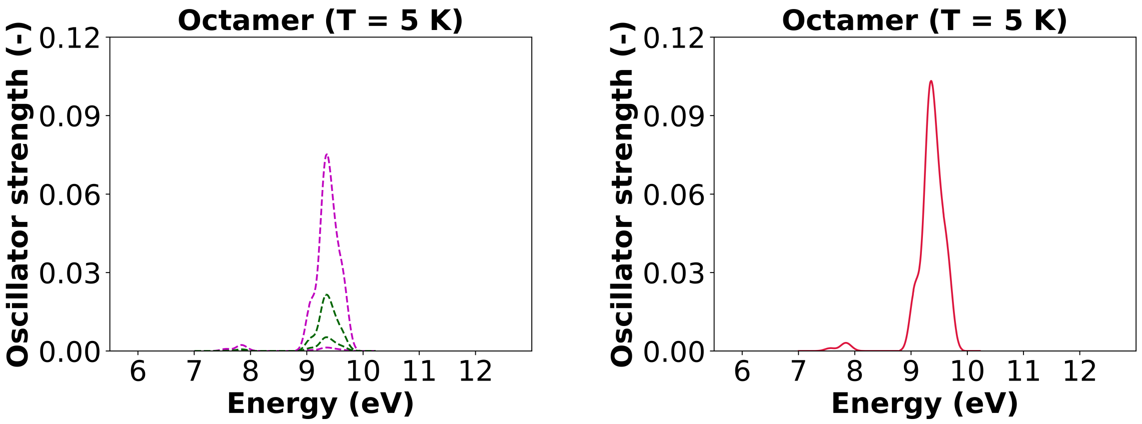

Figure 17: Boltzmann weighting of the **8mer** at 5 K. Left: UV-vis spectrum of each conformer multiplied by the corresponding Boltzmann weight ($\omega > 1\%$). Right: Resulting Boltzmann weighted spectrum.

1.3.9 Larger clusters (16mer/ 24mer/ 48mer)

In addition to the small clusters, the **16mer**, **24mer**, and **48mer** were investigated. As CREST resulted in close to 800 structures or more, and costs for DFT reoptimizations increased with N^2, we only optimized 10 selected conformations, the lowest energy one and 9 further structures. As with increasing cluster size more excitations emerge, that are often dark, the number of nroots was increased to 70.

1.3.10 16mer

For the **16mer** cluster, CREST calculated 903 minima structures for the **16**, but only the lowest four xTB conformers and six additional ones were reoptimized with DFT. As evident from Fig. 18, structural differences between the conformations become increasingly difficult to discern by visual inspection and likewise the spectra show more and more similar UV-vis signatures. Also the relative energy differences become more similar and the six disaplayed structures are within a range of around 20 kJ/mol

The Boltzmann-weighted spectra are shown in Fig. 19, where only the structure with the lowest absolute energy contributes to the Boltzmann spectrum.

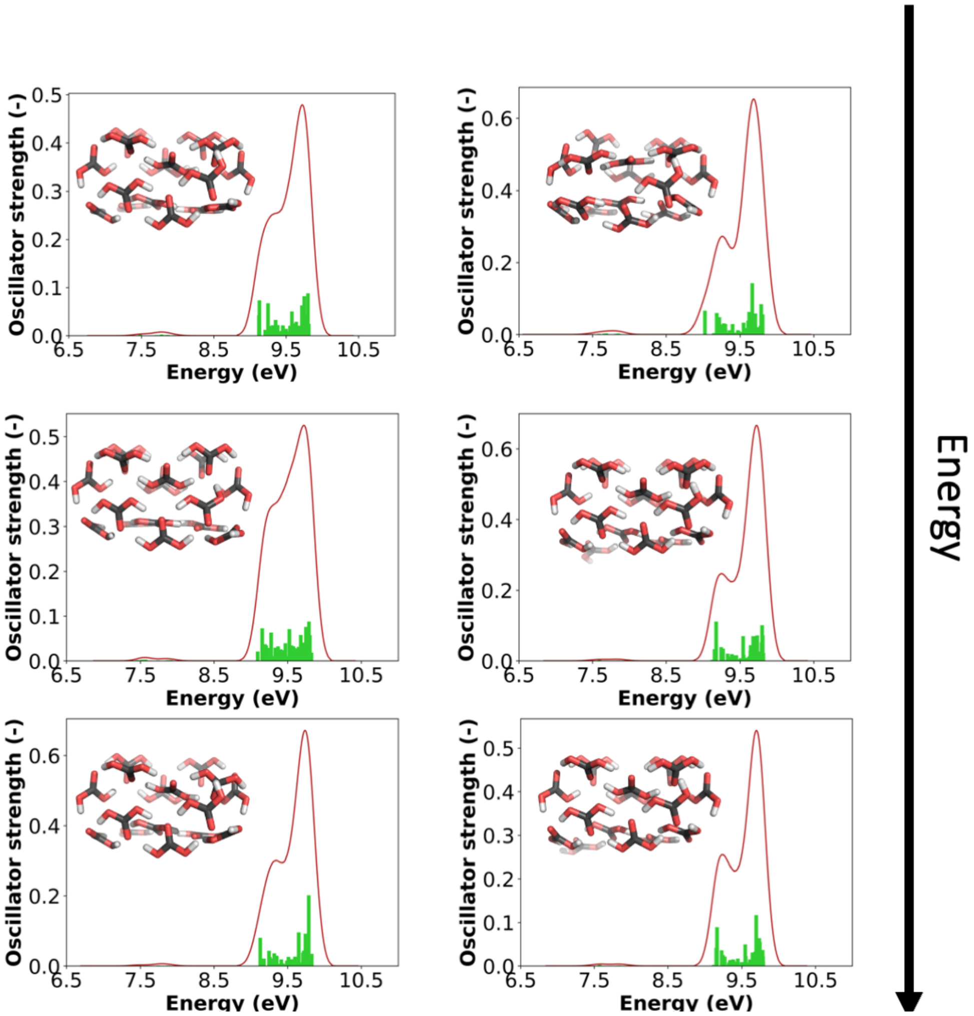

Figure 18: Selected **16mer** conformations and the corresponding UV-vis spectra.

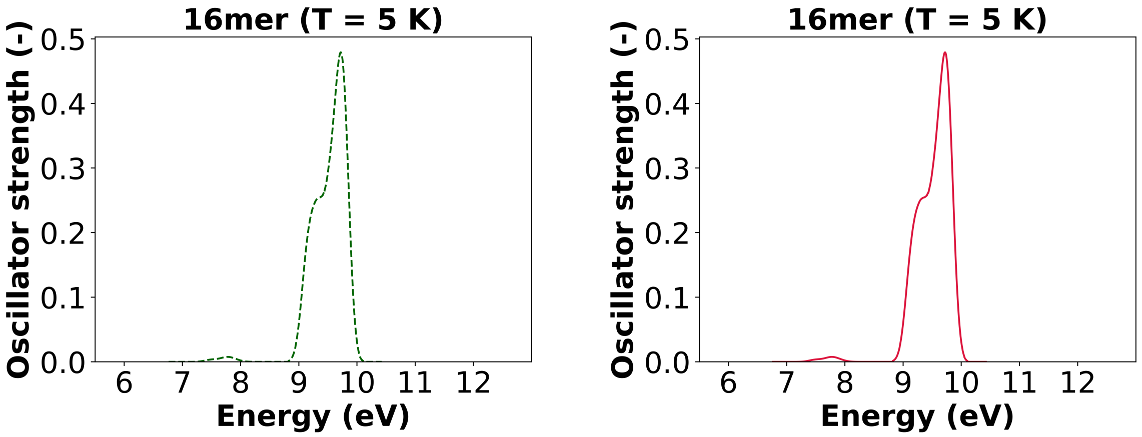

Figure 19: Boltzmann weighting of the **16mer** at 5 K. Left: UV-vis spectrum of each conformer multiplied by the corresponding Boltzmann weight ($\omega > 1\%$). Right: Resulting Boltzmann weighted spectrum.

1.3.11 24mer

CREST identified 782 unique minimum energy conformations for the **24mer**, whereby the lowest four and additional six were reoptimized with full DFT. Compared to the **16mer**, the resulting **24mer** are even more similar, each showing a spherical, non-planar arrangement of monomer units (cf. Fig. 20). Interestingly, a low energy peak at around 7.7 eV emerges. The Boltzmann weighted spectra is shown in 21.

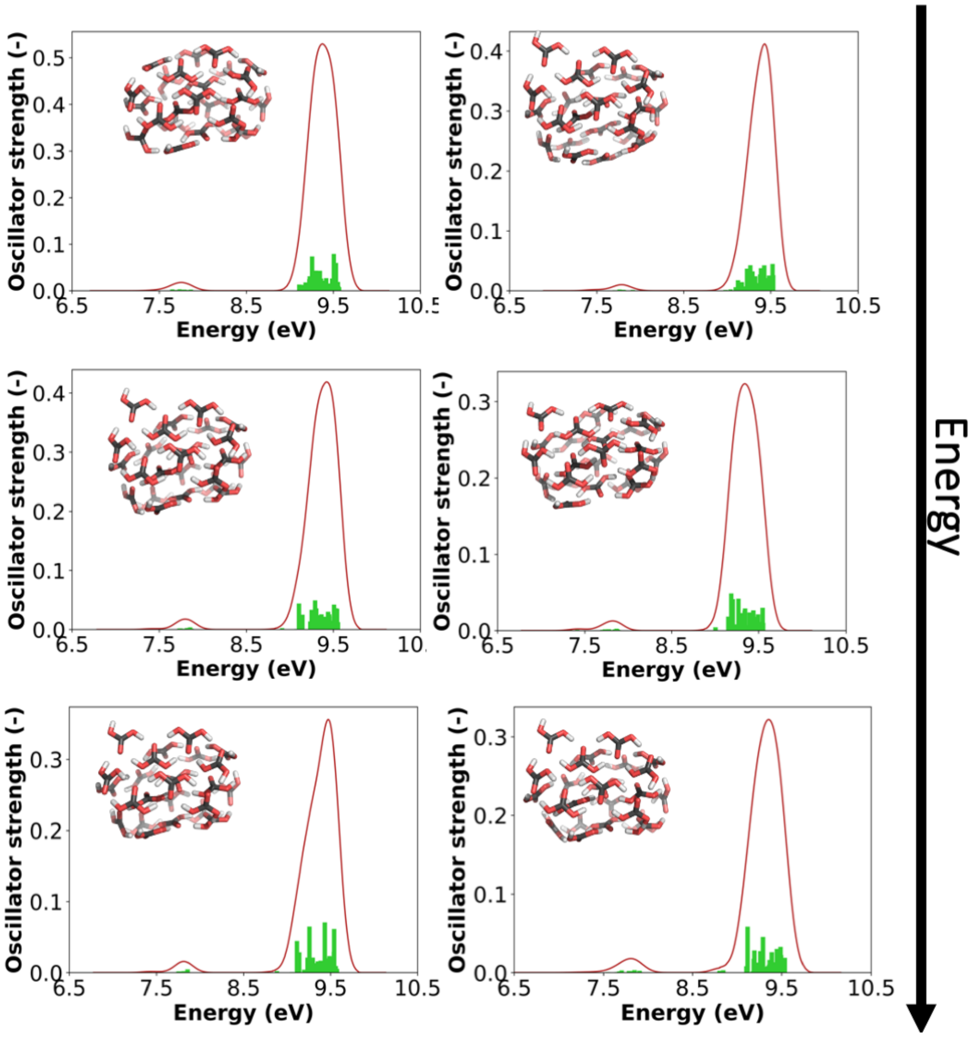

Figure 20: Selected **24mer** minima structures and the corresponding UV-vis spectra.

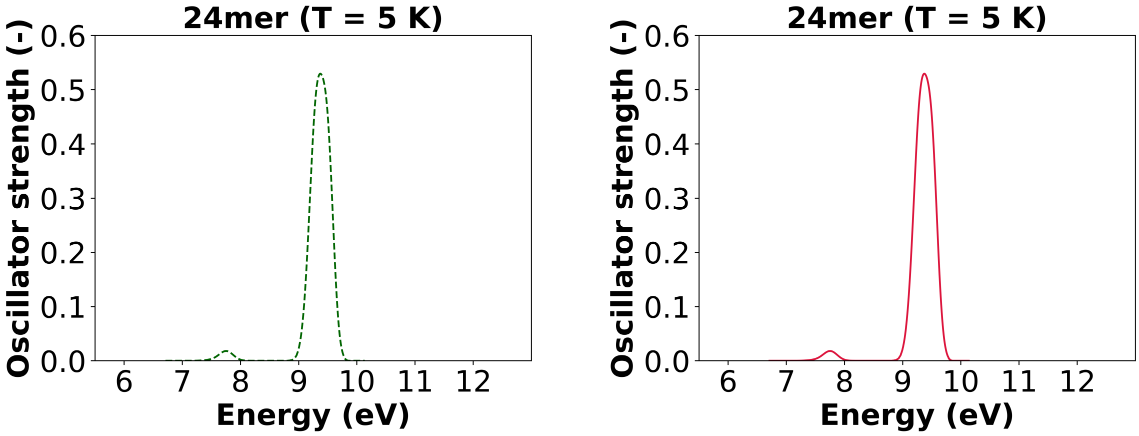

Figure 21: Boltzmann weighting of the **24mer** at 5 K. Left: UV-vis spectrum of each conformer multiplied by the corresponding Boltzmann weight ($\omega > 1\%$). Right: Resulting Boltzmann weighted spectrum.

1.3.12 48mer

For the **48mer**, 216 unique low-energy conformations were detected. From our experience, CREST at times yield a smaller number of structures, potentially because the algorithm was stuck in a deep potential energy minimum. However, to be comparable to the other results, we refrained from changing the CREST settings. In addition, we already saw that resulting UV-vis spectra are very similar for various conformations of the **24mer**. Hence, we used the same protocol as for the smaller clusters and optimized the four lowest energy conformations and additional six that are higher in energy. As visible in Fig. 22, all conformations yield very similar spectra, despite differences in energy of up to 50 kJ/mol. In addition, conformational differences cannot be detected with the naked eye, hence, we only showed Fig. 23 shows the Boltzmann weighted spectra at 5 K.

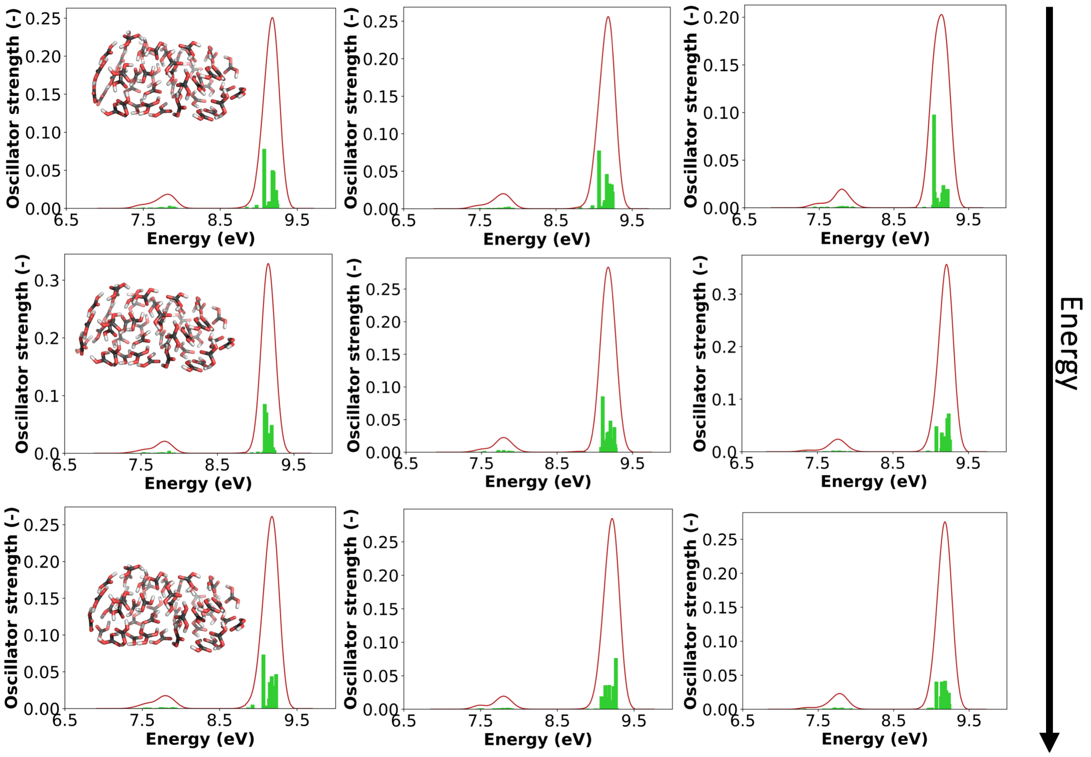

Figure 22: Selected **48mer** minima structures and the corresponding UV-vis spectra. Energy of the conformers increases from top to bottom and left to right.

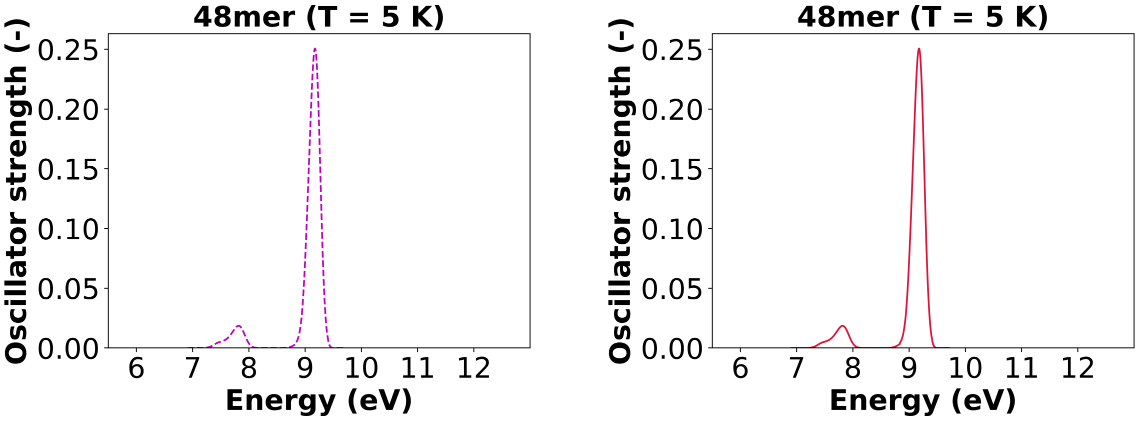

Figure 23: Boltzmann weighting of the **48mer** at 5 K. Left: UV-vis spectrum of each conformer multiplied by the corresponding Boltzmann weight ($\omega > 1\%$). Right: Resulting Boltzmann weighted spectrum.

1.3.13 Comparison of redshift

To compare the redshift across the various cluster sizes, we summarized all Boltzmann weighted spectra in Fig. 24 starting from the **trimer**. While for the **trimer** to **hexamer** a redshift is hardly noticeable, visual redshifts occur starting from the **septamer**, once a nonplanar structure becomes most stable. The plot also highlights the emergence of a low energy peak at around 7.7 eV. These results nicely illustrate the increasing redshift upon cluster size.

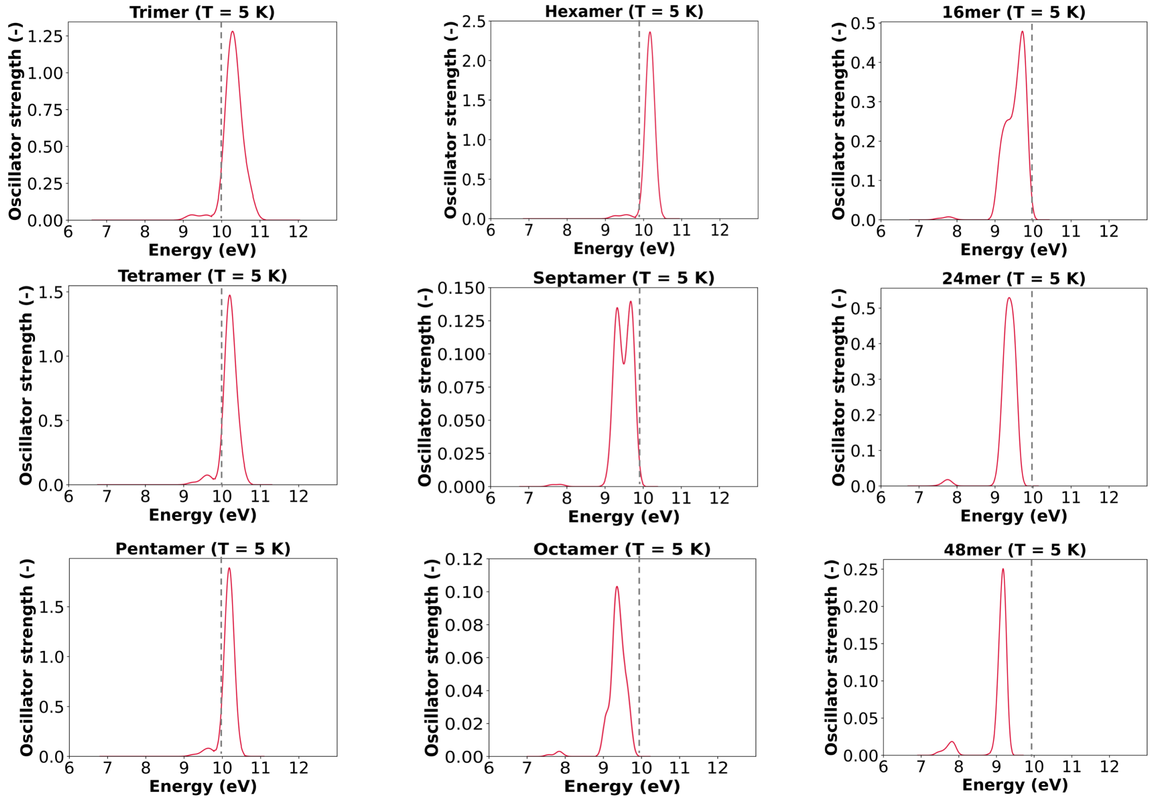

Figure 24: Comparison of Boltzmann weighted UV-vis spectra for the various cluster sizes. A dotted line at 10 eV is added to aid visual comparison.

2 Ordered structural motifs

Slabs of proposed crystal structures of H_2CO_3 were investigated to gauge the effect of increasing size and ordered 3D structural effects on the redshift.

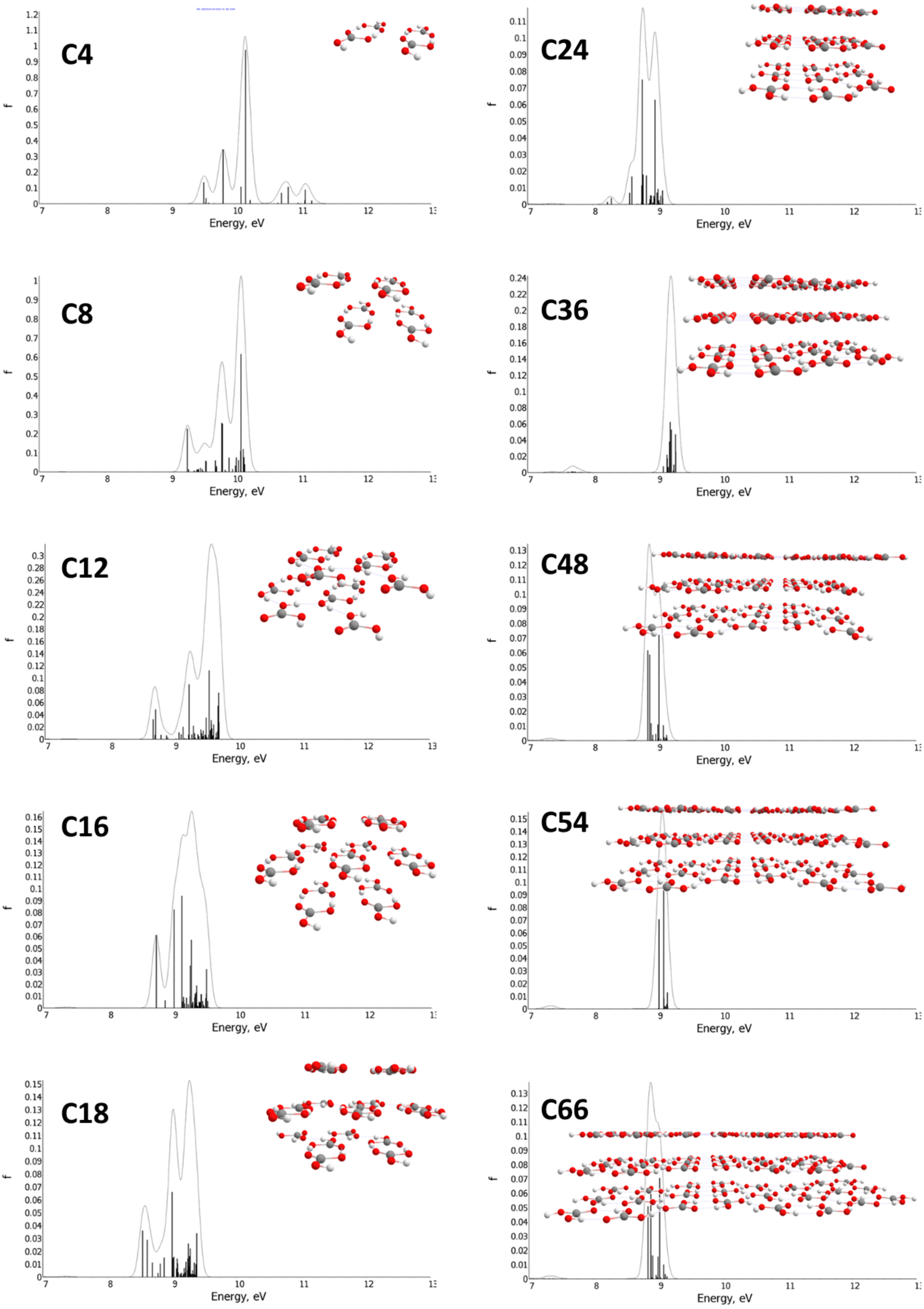

Figure 25: Calculated UV-vis spectra of slabs with increasing carbonic acid units in the computationally proposed H_2CO_3crystal structure **b22**.

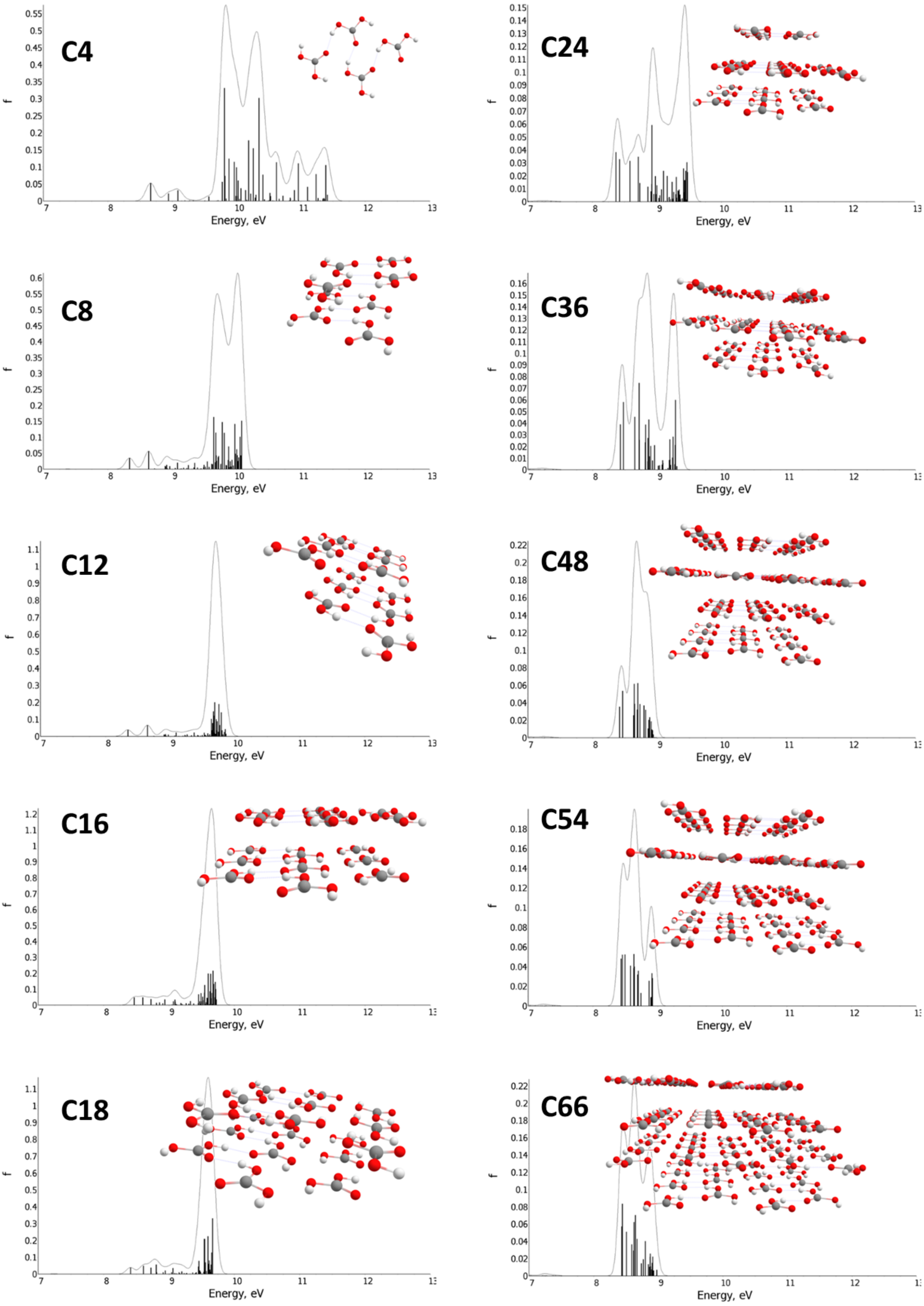

Figure 26: Calculated UV-vis spectra of slabs with increasing carbonic acid units in the computationally proposed H_2CO_3crystal structure **tt_ak11**.

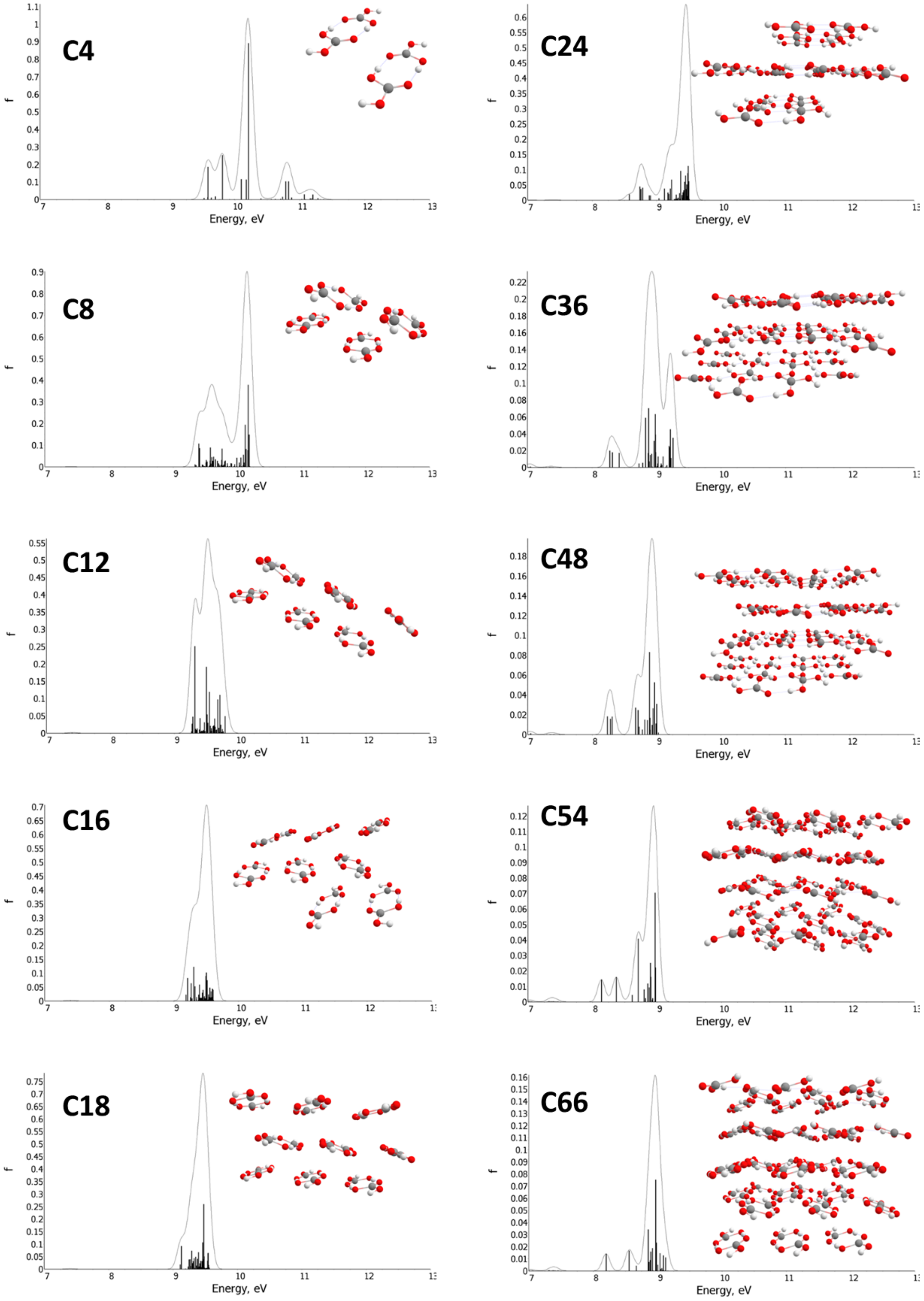

Figure 27: Calculated UV-vis spectra of slabs with increasing carbonic acid units in the computationally proposed H_2CO_3crystal structure **ak35**.

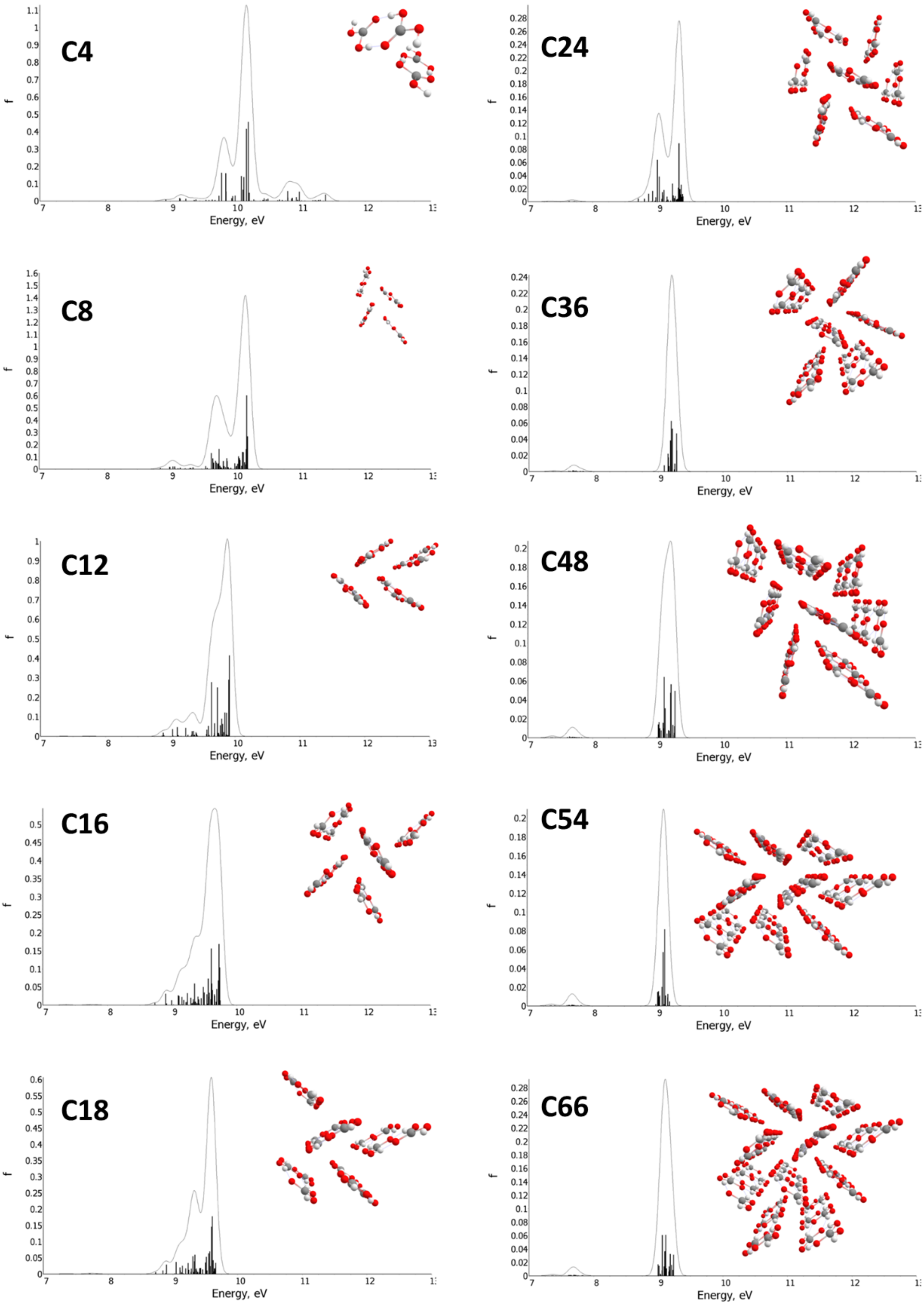

Figure 28: Calculated UV-vis spectra of slabs with increasing carbonic acid units in the computationally proposed H_2CO_3crystal structure **bi8**.

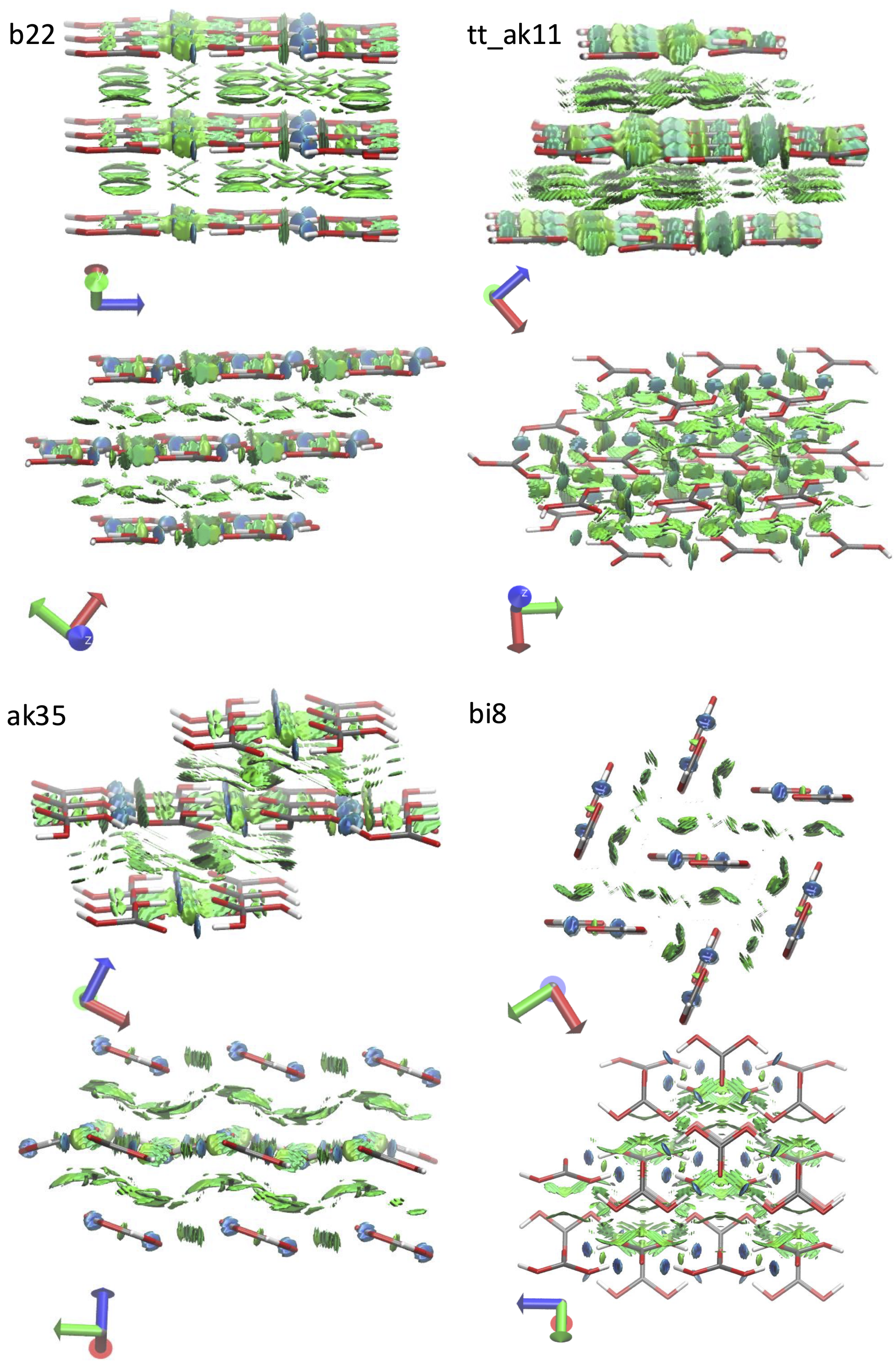

Figure 29: Visualization of noncovalent interactions for the 24mer crystal structure slabs. An isosurface of the reduced electron density gradient of 0.05 a.u. is displayed on which the 2nd eigenvalue of the Laplacian is plotted. It can be used to distinguish strongly attractive noncovalent interactions (depicted in blue), weak, van der Waals like interactions (depicted in green) and repulsive interactions, as arising from steric hindrance (not visible but usually depicted in red).

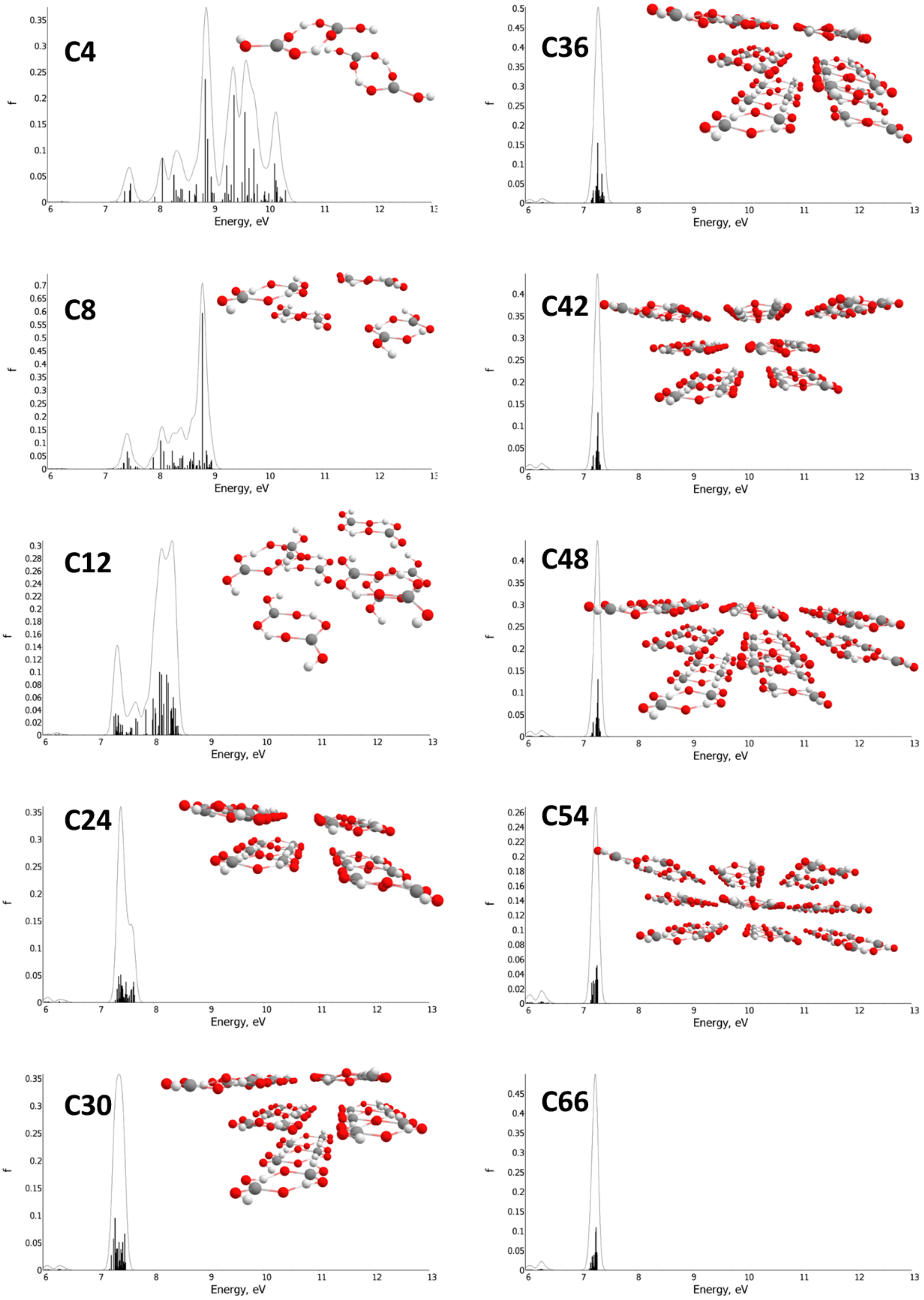

Figure 30: Calculated UV-vis spectra of slabs with increasing carbonic acid units in the experimentally proposed D_2CO_3 high-pressure crystal structure.

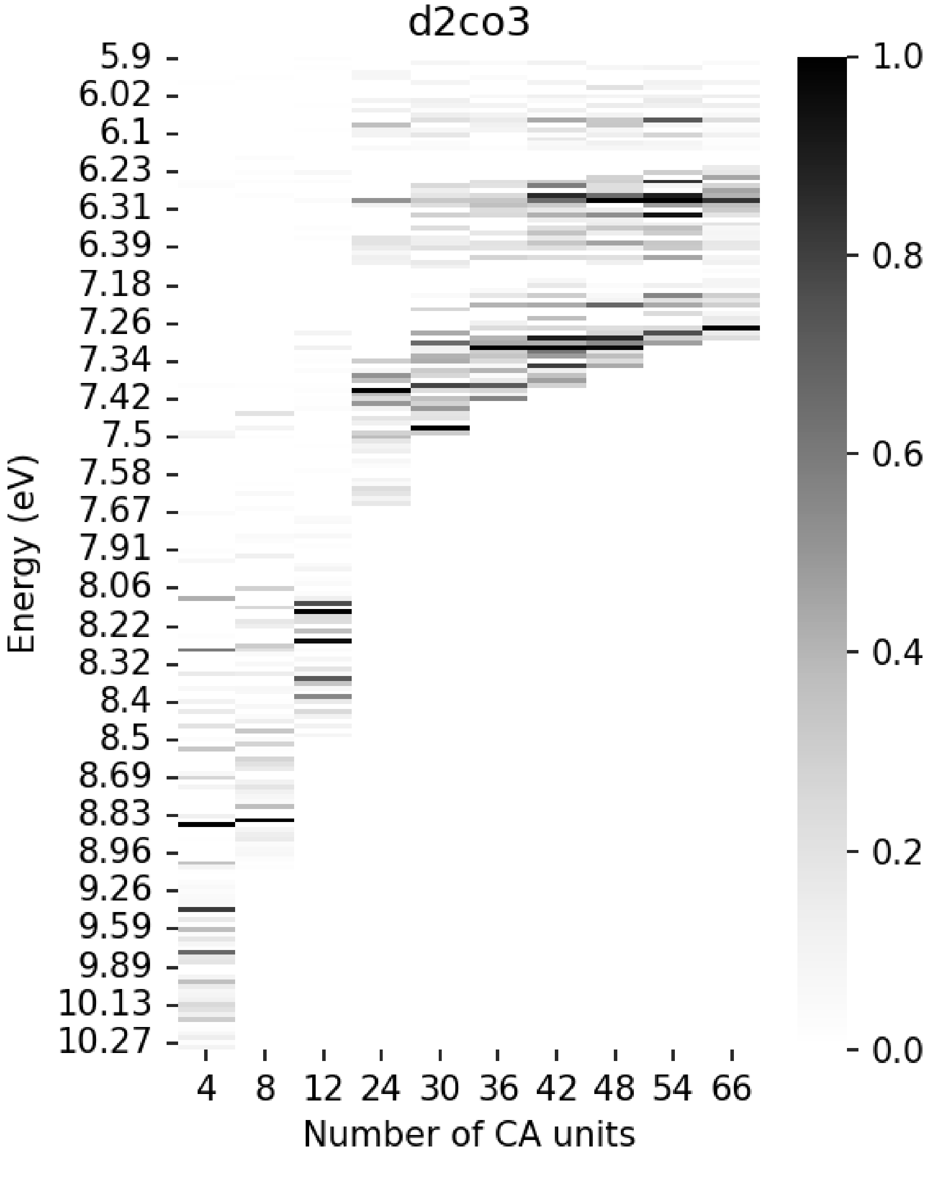

Figure 31: Redshift in the UV-vis spectrum for the ordered clusters of up 66 carbonic acid (CA) units for the experimentally derived high-pressure D_2CO_3 structure.
